# A Compound Screen Based on Isogenic hESC‐Derived β Cell Reveals an Inhibitor Targeting ZnT8‐Mediated Zinc Transportation to Protect Pancreatic β Cell from Stress‐Induced Cell Death

**DOI:** 10.1002/advs.202413161

**Published:** 2025-04-07

**Authors:** Rui Hu, Qing Ma, Yunhui Kong, Zhaoyue Wang, Minglu Xu, Xiangyi Chen, Yajuan Su, Tinghui Xiao, Qing He, Xuan Wang, Wenjun Xu, Yiling Yang, Xushu Wang, Xiaobo Li, Yanfang Liu, Shuangshuang Chen, Rui Zhao, Meng Guo, Gaowei Wang, Weida Li

**Affiliations:** ^1^ Institute for Regenerative Medicine State Key Laboratory of Cardiology and Medical Innovation Center Shanghai East Hospital Frontier Science Center for Stem Cell Research Shanghai Key Laboratory of Signaling and Disease Research School of Life Sciences and Technology Tongji University Shanghai 200092 China; ^2^ Institute of Modern Biology Nanjing University Nanjing 20018 China; ^3^ Department of Pathology Changhai Hospital Navy Medical University Shanghai 200433 China; ^4^ Institute of Translational Medicine Shanghai University Shanghai 200444 China; ^5^ National Key Laboratory of Medical Immunology and Institute of Immunology Navy Medical University Shanghai 200433 China

**Keywords:** isogenic human embryonic stem cell‐derived β cells, stress‐induced cell death, zinc transportation

## Abstract

Pancreatic β cell loss by cellular stress contributes to diabetes pathogenesis. Nevertheless, the fundamental mechanism of cellular stress regulation remains elusive. Here, it is found that elevated zinc transportation causes excessive cellular stress in pancreatic β cells in diabetes. With gene‐edited human embryonic stem cell‐derived β cells (SC‐β cells) and human primary islets, the results reveal that elevated zinc transportation initiates the integrated stress response (ISR), and ultimately leads to β cell death. By contrary, genetic abolishment of zinc transportation shields β cells from exacerbated endoplasmic reticulum stress (ER stress) and concurrent ISR. To target excessive zinc transportation with a chemical inhibitor, an isogenic SC‐β cells based drug‐screening platform is established. Surprisingly, independent of its traditional role as protein synthesis inhibitor at a high‐dose (10 µm), low‐dose (25 nm) anisomycin significantly inhibits zinc transportation and effectively prevents β cell loss. Remarkably, in vivo administration of anisomycin in mice demonstrates protective effects on β cells and prevents type 2 diabetes induced by high‐fat diet. Overall, elevated zinc transportation is identified as a crucial driver of β cell loss and low‐dose anisomycin as a potential therapeutic molecule for diabetes.

## Graphical abstract

Pancreatic β cell loss by cellular stress contributes to diabetes pathogenesis. The study demonstrates that WFS1–ZnT8–zinc axis regulates vicious cycle of zinc transportation and cellular stress in pancreatic β cell. Based on that, the study applies a strategy for compound screening targeting ZnT8 based on human models, and identifies anisomycin, as a ZnT8 chemical inhibitor in β cells at low‐dose.

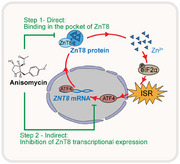

## 1 Introduction

Prevention of the progressive deterioration of therapeutically important cells of human beings is a key challenge for medical intervention for chronic disease. Particularly, pancreatic β cell failure by exacerbated endoplasmic reticulum (ER) stress and coupled integrated stress response (ISR) plays a central role in type 2 diabetes (T2D).^[^ ^1^, ^2^, ^3^, ^4^, ^5^, ^6^, ^7^, ^8^, ^9^ ^]^ Once ER stress and ISR are not alleviated, stress‐induced β cell death occurs. Nevertheless, the underlying mechanism of stress regulation to prevent β cell loss remains elusive, which limits exploration of effective medications. As a result, few of the currently prescribed medications for type 2 diabetes protect β cell mass from stress‐induced cell death in human patients,^[^ ^10^, ^11^ ^]^ resulting in inefficient treatment.

T2D Genome‐wide association studies have uncovered a pivotal causative gene implicated in stress‐induced β cell loss, known as *WFS1*. Deficiency in *WFS1* has been linked to an increased risk of T2D.^[^ ^12^, ^13^ ^]^ Accumulating evidence from rodent studies has shown that loss‐of‐function (LOF) mutations in *WFS1* lead to ER stress disorders and impair β cells, ultimately resulting in diabetes.^[^ ^14^, ^15^ ^]^ Our previous work demonstrated *WFS1*‐LOF disrupts β cell fate trajectory toward maturation and directs it toward stress, ultimately resulting in β cell loss.^[^ ^16^ ^]^ By contrast, *ZNT8*, encoding the insulin granular zinc transporter in pancreatic β cells, exhibits rare genetic LOF mutations that confer protection against diabetes in carriers, suggesting a protective mechanism.^[^ ^12^ ^]^ ZnT8‐LOF has been shown to enhance insulin secretion,^[^ ^12^, ^17^, ^18^, ^19^ ^]^ predicting chemical inhibition of zinc transportation holds potential to protect β cells. However, the intricate interplay between WFS1, ZnT8‐mediated zinc transportation, and stress‐induced β cell failure remains elusive, thereby hindering the development of therapeutic approaches aimed at protecting β cells through chemical inhibition of ZnT8.

Furthermore, lack of proper human model limits drug discovery for ZnT8 chemical inhibitor. Because of significant interspecies differences,^[^ ^20^, ^21^ ^]^ numerous drug candidates identified from disease models based on experimental animals or cell lines are often not applicable to human patients, contributing to poor success rates (≈10%) and substantial costs.^[^ ^22^, ^23^ ^]^ This underscores the pressing need for proper human models in drug discovery.^[^ ^24^ ^]^ Recently, the FDA has approved the application of human organoids to replace traditional mouse models, heralding a novel approach to drug discovery. Of particular significance are human embryonic stem cell derived β cells (SC‐β cells), holding great promise for drug discovery efforts aimed at preserving β cell mass during diabetes pathogenesis.^[^ ^25^, ^26^ ^]^

**Figure 1:**
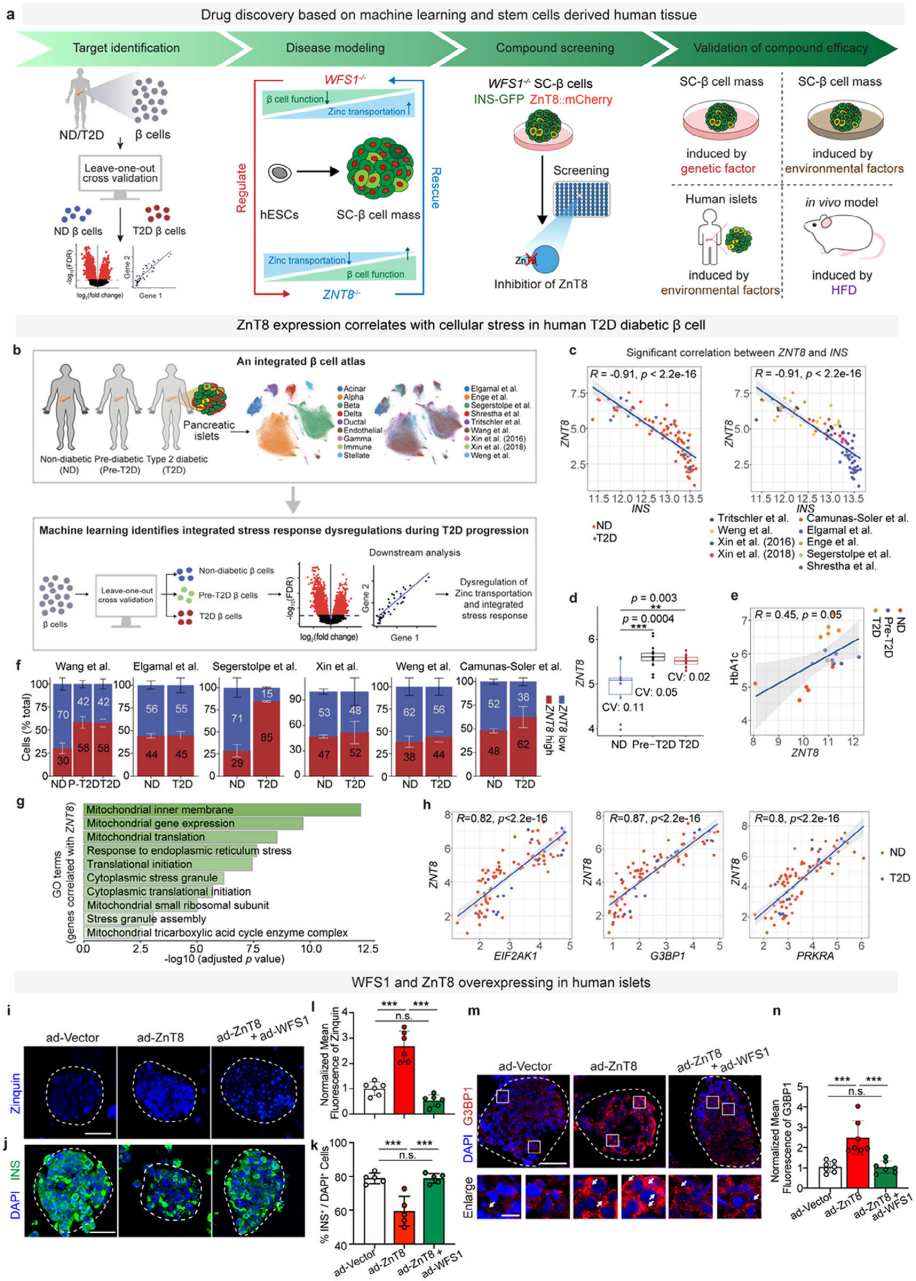
Elevated zinc transportation causes excessive cellular stress in pancreatic β cells in diabetes. a) Schematic diagram of drug discovery based on stem cell‐derived human tissue. b) Schematic diagram of the data collection and data analysis. c) Pearson correlation between *ZNT8* and *INS* expression level across donors. These donors are from distinct disease stage (left) and cohort (right). Gene expression level was transformed using ln (TPM +1). d) Changes of *ZNT8* expression level from ND to Pre‐T2D, and T2D β cells^[^ ^28^ ^]^ (ANOVA test with age, sex, BMI, and islet index as covariates). e) Pearson correlation between *ZNT8* expression level and HbA1c across donors. Gene expression level were transformed using ln(TPM +1). f) Composition shift of *ZNT8* high and *ZNT8* low β cell subpopulation in six independent cohorts. *ZNT8* high and *ZNT8* low β cell subpopulation were defined based on median ZNT8 expression level in β cell for individual cohorts. g) Enrichment analysis of genes that exhibit significant positive correlation with *ZNT8*. h) Pearson correlation between *ZNT8* and *EIF2AK1*, *G3BP1*, *PRKRA* expression level across donors. These donors are from distinct disease stage. Gene expression level has been transformed using ln(TPM +1). i) Representative images of fluorescence of Zinquin (blue) in human primary islets transduced with adenoviral (ad) vectors, including ad‐Vector, ad‐ZnT8, and ad‐ZnT8+ad‐WFS1, islets are marked with white dotted circles. l) Quantification of mean fluorescent intensity of Zinquin in (i) (one‐way ANOVA, *n* = 6) (normalized with ad‐Vector transduced group)). j) Representative images of immunofluorescent staining of insulin (green) and DAPI (blue) of human primary islets transduced with including ad‐Vector, ad‐ZnT8, and ad‐ZnT8+ad‐WFS1, islets are marked with white dotted circles. k) Quantification of INS^+^ β cells in (j) (one‐way ANOVA, *n* = 5 (normalized with ad‐Vector transduced group)). m) Representative images of immunofluorescent staining of G3BP1 (red) and DAPI (blue) of human primary islets transduced with including ad‐Vector, ad‐ZnT8, and ad‐ZnT8+ad‐WFS1, islets are marked with white dotted circles. n) Quantification of mean fluorescent intensity of G3BP1 in (m) (one‐way ANOVA, *n* = 7 (normalized with ad‐Vector transduced group)). Scale bars: 50 µm (i,k,m). Data are mean ± s.d. Individual data points are shown for all bar graphs. *P < 0.05; **P < 0.01; ***P < 0.001; n.s., not significant.

Aiming to investigate the pathological mechanism of how cellular stress is regulated in pancreatic β cell in diabetic conditions and explore novel therapeutic molecules to preserve pancreatic β cell mass in diabetes, we applied a strategy for drug discovery based on a human model including identification of novel therapeutic target, disease modeling, compound screening, and validation of drug efficacy (**Figure** **1**a). Our findings reveal that low‐dose anisomycin, as a ZnT8 inhibitor, protects against cellular stress‐induced cell death, thereby safeguarding β cells both in vitro and in vivo.

## 2 Results

### 2.1 Identification of Zinc Transportation Induced Cellular Stress in Human Diabetic β Cells

Previous studies showed that differentially expressed genes (DEGs) between nondiabetic and type 2 diabetic β cell are not robust across cohorts.^[^ ^27^ ^]^ Lack of reliable DEGs limits therapeutic targets, driving pathomechanism and drug discovery for effective medications for T2D. To this end, we first collected publicly available islet single‐cell data, resulting in an integrated human β cell atlas that include 71178 nondiabetic cells (from 112 donors) and 30 021 type 2 diabetic β cells (from 48 donors) (Table S1, Supporting Information). To address the heterogeneity challenge, we used a machine learning algorithm we previously proposed to analyze human islet single‐cell data^[^ ^28^ ^]^ (Figure 1b) and identified 31 consistently and robustly changed genes across independent cohorts (Table S2, Supporting Information). Among these DEGs between nondiabetic and type 2 diabetic β cells, we found the downregulation of *INS* (Figure S1a, Supporting Information) as expected. With the integrated human β cell atlas, we identified genes with significant correlation with *INS*. Among these, we validated that another robustly changed gene, *ZNT8*, which encodes the insulin granular zinc transporter ZnT8, exhibits a significant negative correlation with *INS* across disease status and cohorts (Figure 1c; Figure S1b, Supporting Information). Moreover, reanalyzing data from our previous study^[^ ^28^ ^]^ that include β cells from nondiabetic, prediabetic, and type 2 diabetic donors, we found *ZNT8* increased significantly from nondiabetic to prediabetic β cells and type 2 diabetic β cells (Figure 1d; Figure S1c, Supporting Information). Of note, the decreased coefficient of variation of *ZNT8* from nondiabetic to type 2 diabetic donors further suggest the convergence of ZnT8 overexpression (O.E) during type 2 diabetes progression (Figure 1d). We also found positive correlation between HbA1c level (an index for long‐term glycemic control) and β cell *ZNT8* expression level of individual donors (Figure 1e). We further confirmed our conclusions by classifying β cells from six independent cohorts as *ZNT8* low and *ZNT8* high subpopulation using median expression level of *ZNT8* across β cells. Results showed that proportion of *ZNT8* high subpopulation increased in all the cohorts of T2D diabetic patients (Figure 1f). To further explore the biological processes associated with ZnT8, we identified positively correlated genes with *ZNT8* across disease status and cohorts using the integrated β cell atlas. Enrichment analysis using these positively correlated genes suggested that upregulated ZnT8 is relevant to mitochondrial dysfunctions and cytoplasmic stress (Figure 1g). For example, ISR^[^ ^29^ ^]^ relevant genes *G3BP1*, *EIF2AK1*, *PRKRA* exhibit significant positive correlation with *ZNT8* (Figure 1h; Figure S1d, Supporting Information). Since *ZNT8* encodes β cell specific zinc transporter, results from the integrated human β cell atlas suggested elevated zinc transportation may play a causal role in cellular stress induced β cell loss in type 2 diabetes.

Previous GWAS data across multiancestry populations identified significant associations between zinc transporter (ZnT8) variants and T2D risk, which revealed that the rare LOF variants of *ZNT8* protect against diabetes in human subjects.^[^ ^17^, ^18^ ^]^ Furthermore, by analyzing T2D risk variants and chromatin accessibility of the *ZNT8* gene body and proximal regulatory regions, we found T2D risk variants (e.g., rs13266634) associated with changes in β cell chromatin accessibility of cis‐regulatory elements of *ZNT8* (Figure S1e, Supporting Information). Consistently, these human GWAS data also suggest the causal role of *ZNT8* dysregulation in driving T2D progression.

To further validate ZnT8 as a therapeutic target for human islet β cell loss and examine its interaction with WFS1 (reported to prevent β cell loss from ER stress and ISR), we overexpressed ZnT8, WFS1, or both in primary human islets via adenovirus (with CMV promoter) transduction to examine β cell mass labeled by Zinquin staining (zinc dye for living islets) or insulin immunostaining. Surprisingly, as compared to control, O.E WFS1 significantly suppressed zinc transportation in human islets, downregulated expression of G3BP1 and attenuated stress granule formation, but increased β cell population. In parallel, O.E ZnT8 significantly increased zinc transportation, upregulated expression of G3BP1 and promoted stress granule formation, but significantly reduced β cell population. By contrast, the reduction of β cell population by O.E ZnT8 was reversed when ZnT8 was co‐overexpressed with WFS1 (Figure 1i–n).

Altogether, these results from human samples identified upregulated ZnT8 mediated zinc transportation in T2D as a therapeutic target for β cell stress and loss, and implied the negative regulation of ZnT8 mediated zinc transportation by WFS1 as a potential protective mechanism to preserve human β cell mass against stress‐induced cell death.

### 2.2 *WFS1* Deficiency Results in Elevated Zinc Transportation and Consequent Stress Granule Formation in Human SC‐β Cell Mass

**Figure 2:**
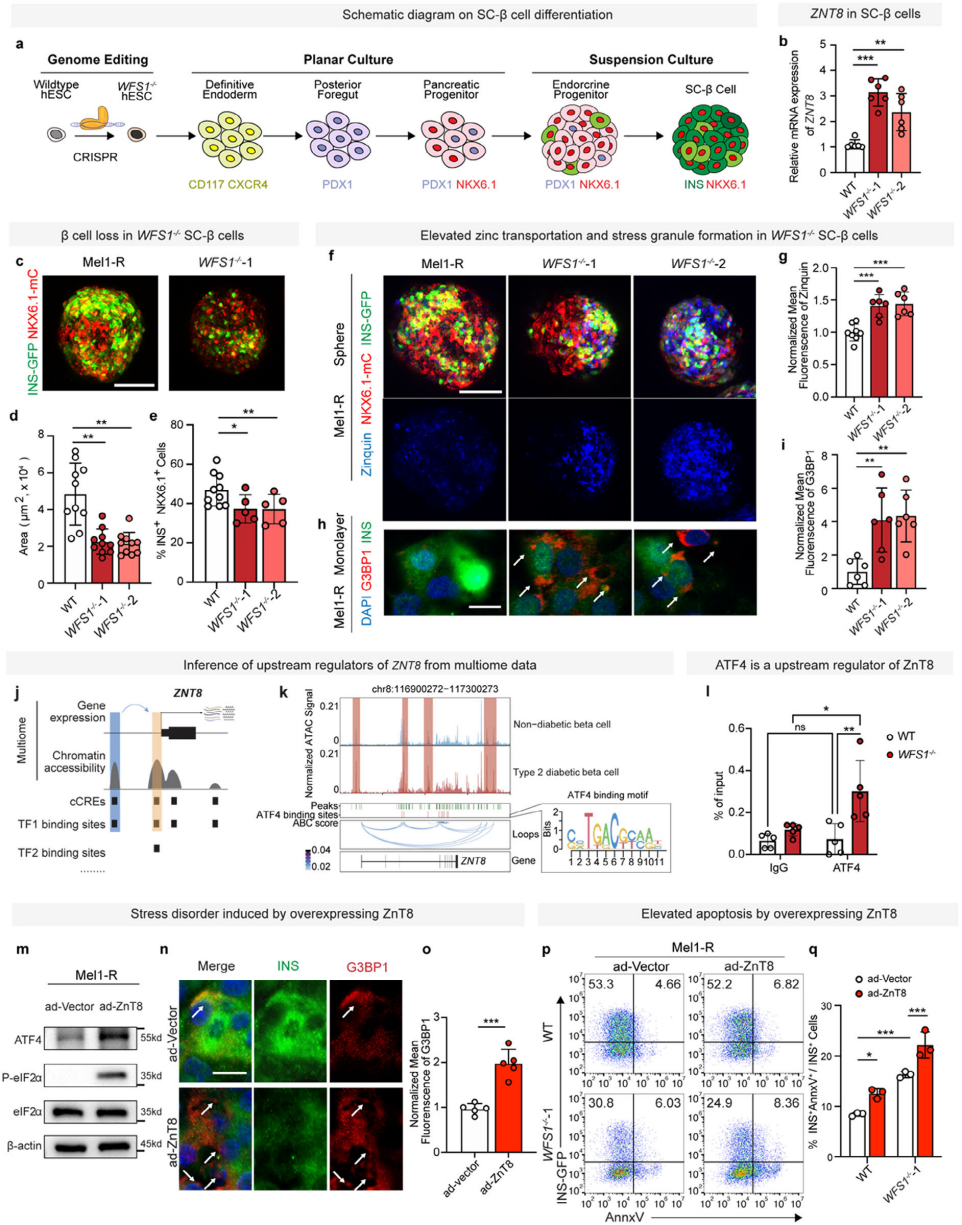
*WFS1* deficiency results in elevated zinc transportation and consequent stress granule formation in human SC‐β cell mass. a) Schematic diagram of the differentiation protocol used for SC‐β cell differentiations. Bottom row: key markers of every stage (DE: *CD117* and *CXCR4*; PF: *PDX1*; PP, EP: *PDX1* and *NKX6.1*; SC‐β cells: *INS* and *NKX6.1*). GFP, green fluorescent protein; mC, mCherry. b) qPCR of *ZNT8* of wild‐type and *WFS1^−/−^* SC‐β cells on D10 at the SC‐β cell stage (one‐way ANOVA, *n* = 6) (normalized with WT group). c) Representative images of wild‐type (WT) and *WFS1^−/−^* SC‐β cell clusters on D10 at the SC‐β cell stage. d) Quantification of spheroid sizes (in square micrometers) of WT and *WFS1^−/−^* SC‐β cell on D10 at the S7 stage (one‐way ANOVA, *n* = 10). e) Representative flow cytometry dot plots showing the percentage of β cell population (INS‐GFP^+^NKX6.1‐mCherry^+^) of WT and *WFS1^−/−^* SC‐β cell on D10 at the S7 stage (one‐way ANOVA, *n* = 10 (WT), 5 (*WFS1^−/−^*‐1), 5 (*WFS1^−/−^*‐2)). f) Representative images of fluorescence of clusters of WT and *WFS1^−/−^* SC‐β cell on D10 at the S7 stage of the protocol with Zinquin (blue). g) Quantification of mean fluorescent intensity of Zinquin in (f) (one‐way ANOVA, *n* = 8 (WT), 6 (*WFS1^−/−^*‐1), 6 (*WFS1^−/−^*‐2)) (normalized with WT group). h) Representative images of fluorescence of G3BP1 (red), INS (green), and DAPI (blue) in adherent single cell dissociated from WT and *WFS1^−/−^* SC‐β cell on D10 at the S7 stage. i) Quantification of mean fluorescent intensity of G3BP1 in (h) (one‐way ANOVA, *n* = 6) (normalized with WT group). j) Schematic diagram of the inference of upstream regulator of *ZNT8*. k) Genome browser tracks at *ZNT8* gene showing aggregate ATAC read density in ND and T2D β cell, β cell candidate cis‐regulatory elements (cCREs), β cell cCREs with ATF4 binding sites, β cell Hi‐C interactions loops. cCREs with increased chromatin accessibility in T2D β cells compared to ND β cells were highlighted. l) Relative expression of ZNT8 mRNA in WT and *WFS1^−/−^* SC‐β cell on D10 at the S7 stage. Enrichment of DNA bound to ATF4 antibody (two‐way ANOVA, *n* = 5). m) Western blots of ATF4, eIF2α, P‐eIF2α, and β‐actin of WT SC‐β cells transduced with ad‐Vector and ad‐ZnT8 for 48 h. n) Representative images of fluorescence of G3BP1 (red), INS (green), and DAPI (blue) of WT SC‐β cells transduced with ad‐Vector and ad‐ZnT8 for 48 h. o) Quantification of mean fluorescent intensity of G3BP1 in (n) (unpaired two‐tailed *t‐*tests, *n* = 5) (normalized with ad‐Vector transduced group). p) Representative flow cytometry dot plots showing the percentage of apoptotic β cells (INS‐GFP^+^AnnxV^+^/ INS‐GFP^+^) induced by ad‐Vector and ad‐ZnT8 in WT and *WFS1^−/−^*‐1 SC‐β cells. q) Flow cytometry quantification of apoptotic β cells in (p) (two‐way ANOVA, *n* = 3 of each group). Scale bars: 100 µm (c,f), 10 µm (h,n). Data are mean ± s.d. Individual data points are shown for all bar graphs. *P < 0.05; **P < 0.01; ***P < 0.001; n.s., not significant.

Scarcity of human islets samples limits traditional exploration of the therapeutical opportunity of ZnT8. Fortunately, recent human embryonic stem cell (hESC)‐based genome editing and SC‐β cell from pancreatic step‐wise differentiation provide an efficient and reliable genotype to phenotype human model^[^ ^30^, ^31^, ^32^ ^]^ (**Figure** **2**a). In order to investigate pathogenic role of zinc transportation in stress induced cell death of β cell, we modeled that with WFS1‐LOF SC‐β cell, which represents the classic model of stress induced cell death by genetic lesion in T2D and Wolfram syndrome.^[^ ^12^, ^13^, ^33^, ^34^ ^]^ Notably, by comparing DEGs between human type 2 diabetic β cells and nondiabetic β cells, as well as between *WFS1* knockout and wild‐type (WT) SC‐β cells from our previous studies,^[^ ^16^, ^28^ ^]^ we identified 223 genes that were significantly altered in both SC‐β and primary β cells. Subsequent gene ontology (GO) enrichment analysis confirmed that SC‐β cells recapitulate the dysfunctions observed in actual diabetic β cells, including impairments in cytoplasmic translation, intracellular transport, and cytoplasmic stress responses (Figure S1f, Supporting Information).

Moreover, we found that knockout *WFS1* in hESC lines could recapitulate ZnT8 overexpression in human T2D β cells. There were almost threefold increase of *ZNT8* mRNA level in both *WFS1^−/−^* (*WFS^−/−^*‐1 and *WFS^−/−^*‐2) SC‐β cells, compared to WT SC‐β cells (Figure 2b; Figure S2a, Supporting Information). Similar to O.E ZnT8 in WT β cells, the *WFS1^−/−^* SC‐β cell showed reduced size, declined β cell population (Figure 2c–e, Supporting Information), but displayed increased cytosolic calcium, mitochondrial depolarization, and ER stress (Figure S2b–f, Supporting Information). Surprisingly, we also observed significantly elevated zinc transportation with consequent excessive cellular zinc and upregulated expression of G3BP1 representing elevated ISR in *WFS1^−/−^* SC‐β cells (Figure 2f–i, Supporting Information). These results suggested that WFS1‐LOF leads to upregulated ZnT8 expression and elevated zinc transportation, which directly contributes to cellular stress induced β cell loss.

Since we observed a dramatic increase of zinc transportation and upregulation of ZnT8 inside *WFS1^−/−^* SC‐β cells, we wondered how ZnT8 is upregulated in *WFS1^−/−^* SC‐β cells. Consistent with previous reports in SC‐β cells,^[^ ^33^, ^35^ ^]^ *WFS1* deficiency upregulates expression of ATF4 (a stress responsive transcriptional factor mediating gene expression reprogramming during ISR) (Figure S2b, Supporting Information), leading us to hypothesize that ATF4 might activate transcriptional expression of ZnT8 encoded by *ZNT8*. By integrating human islet single‐cell multiomics data and transcription factor (TF) binding dataset^[^ ^28^, ^36^, ^37^ ^]^ (Figure 2j), we found that ATF4 binds to the regulatory elements (promoter and enhancer) of *ZNT8* (Figure 2k) and significant positive correlation between ATF4 and *ZNT8* (Figure S2g, Supporting Information). In fact, we found that chromatin accessibility of some regulatory elements of *ZNT8* are increased in T2D β cells (Figure 2k), indicating that the upregulation of ZnT8 in T2D β cells is already determined at epigenetic level. So, ATF4 links WFS1 and ZnT8 via transcriptional regulations. We then performed ChIP‐qPCR to confirm the binding, and the result showed high ATF4 DNA binding to upstream region of *ZNT8* in *WFS1^−/−^* SC‐β cells (Figure 2l). Increased protein level of ZnT8 by *WFS1* deficiency was also confirmed by Mel1‐ZnT8::mCherry reporter line (*INS^w/GFP^ ZnT8::mCherry*), in which mCherry was fused to the C‐terminus of ZnT8 (Figure S2h,i, Supporting Information). Furthermore, ISRIB (reported to suppress ISR and reduce expression of ATF4^[^ ^38^ ^]^) treatment significantly inhibited zinc transportation in *WFS1^−/−^* SC‐β cells (Figure S2j,k, Supporting Information). These results demonstrated that WFS1‐LOF upregulates ZnT8 mediated zinc transportation by heightened expression of cellular stress responsive transcriptional factor ATF4, and inhibition of ATF4 with ISRIB treatment significantly increased zinc transportation bridging to cellular stress.

It is also reported that mitochondrial dysfunction activates ISR via the eIF2α kinase GCN2,^[^ ^39^, ^40^ ^]^ indeed, we observed that intracellular zinc level, phosphorylation of eIF2α, and expression of G3BP1 were upregulated along with increased cell death of SC‐β cell as a result of overexpressing ZnT8 (Figure S2l,m, Supporting Information; Figure 2m–q). O.E ZnT8 in WT SC‐β cells also elevated ER stress as scored by qPCR of ER stress related genes’ transcriptional expressions (Figure S2n, Supporting Information) and sXBP1 immunostaining. As ER stress increases, XBP1 is spliced into sXBP1 by IRE1a, then sXBP1 protein translocates to nucleus.^[^ ^41^, ^42^ ^]^ We indeed observed significantly increased sXBP1 level in nucleus when zinc transportation was elevated by O.E ZnT8 (Figure S2o,p, Supporting Information).

Besides genetic lesions represented by WFS1‐LOF, environmental insults like lipotoxicity and glucotoxicity also contributes to cellular stress, we simulated these metabolic stress via treatment with palmitic acid (PA, 1 mm), stearic (SA, 0.6 mm), linoleic acid (LA, 0.3 mm), and d‐glucose (35 mm)^[^ ^2^, ^32^, ^43^, ^44^ ^]^ in *WFS1^−/−^* SC‐β cells, and the zinc transportation was significantly elevated, indicating a closer correlation between zinc transportation and cellular stress (Figure S3a,b, Supporting Information).

At the same time, we added extra zinc (200 µm) into the medium to increase zinc transportation in SC‐β cells, resulting in increased cytosolic calcium (Figure S4a–d, Supporting Information). To investigate the relationship between elevated zinc transportation and mitochondria dysfunction, we costained the wild‐type SC‐β cells with Zinquin and MitoTracker Red to visualize intracellular zinc distribution and mitochondrial fragmentation. After excessive zinc (500 µm ZnSO_4_) treatment, zinc colocalized with mitochondria along with abnormal mitochondrial fission dynamically over time within 900 s (Figure S4e–g, Supporting Information), which is consistent with previous report that increase of mitochondrial zinc results in abnormal mitochondrial fission and β cell death in rodents.^[^ ^45^ ^]^ Elevation of zinc transportation by adding extra zinc also increased zinc colocalization with mitochondria and mitochondria depolarization (Figure S4h–k, Supporting Information). The protein DRP1, required for mitochondrial division, was upregulated in zinc‐treated SC‐β cell, and dividing mitochondrial makes contact with lysosome (Figure S4l,m, Supporting Information), indicating the divided mitochondrial portion is degraded.^[^ ^46^, ^47^ ^]^ Moreover, elevated zinc transportation also led to increased stress granule formation (Figure S4n,o, Supporting Information) and cell death in both WT and *WFS1^−/−^* SC‐β cells (Figure S4p–s, Supporting Information). Taken together, these results suggested that elevated cellular stress caused by WFS1‐LOF leads to ATF4‐activated upregulation of ZnT8 expression and elevated zinc transportation. Moreover, elevated zinc transportation directly exacerbates ER stress and ISR, resulting in β cell loss.

### 2.3 Inhibition of Zinc Transportation by Genetic Depletion of ZnT8 Expression Protects β Cell Mass

**Figure 3:**
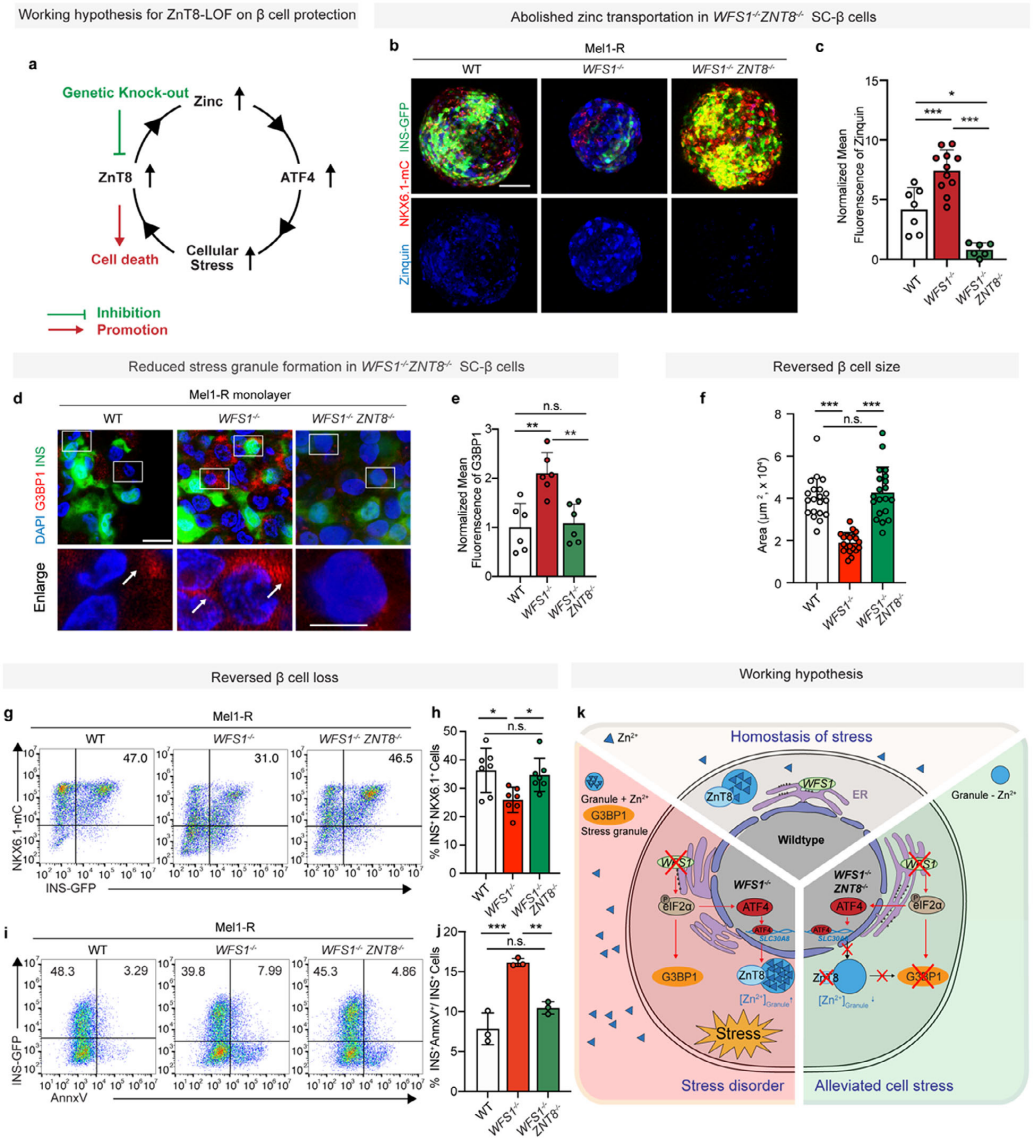
Inhibition of zinc transportation by genetic depletion of ZnT8 expression protects β cell mass from cellular stress. a) Working hypothesis for ZnT8‐LOF on β cell protection. b) Representative images of fluorescence of Zinquin (blue), NKX6.1‐mCherry (red) and INS‐GFP (green) in wild‐type, *WFS1^−/−^* and *WFS1^−/−^ZNT8^−/−^* SC‐β cells on D10 at the S7 stage. c) Quantification of mean fluorescent intensity of Zinquin in (b) (one‐way ANOVA, *n* = 7(WT), 11(*WFS1^−/−^*), 6(*WFS1^−/−^ZNT8^−/−^*)) (normalized with *WFS1^−/−^ZNT8^−/−^* group). d) Representative images of fluorescence of G3BP1 (red), INS (green), and DAPI (blue) in adherent single cell dissociated from WT, *WFS1^−/−^*, and *WFS1^−/−^ZNT8^−/−^* SC‐β cell on D10 at the S7 stage. e) Quantification of mean fluorescent intensity of G3BP1 in (d) (one‐way ANOVA, *n* = 6) (normalized with WT group). f) Quantification of spheroid sizes (in square micrometers) at the SC‐β cell stage (one‐way ANOVA, *n* = 20). g) Representative flow cytometry dot plots of wild‐type, *WFS1^−/−^*, and *WFS1^−/−^ZNT8^−/−^* on D10 at the SC‐β cell stage measuring β cell population (INS‐GFP^+^ NKX6.1‐mCherry^+^). h) Flow cytometry quantification of β cell population (INS‐GFP^+^NKX6.1‐mCherry^+^) in (g) (one‐way ANOVA, *n* = 7). i) Representative flow cytometry dot plots of wild‐type, *WFS1^−/−^* and *WFS1^−/−^ ZNT8^−/−^* on D10 at the SC‐β cell stage measuring apoptotic β cell population (INS‐GFP^+^AnnxV^+^/INS‐GFP^+^). j) Flow cytometry quantification of apoptotic β cell population (INS‐GFP^+^AnnxV^+^ / INS‐GFP^+^) in (i) (one‐way ANOVA, *n* = 3). k) Working hypothesis of mechanism by ZnT8‐LOF alleviates the cellular stress, deduced form the data presented in this study. Scale bar: 100 µm (b), 10 µm (d, upper), 5 µm (d, lower). Data are mean ± s.d. Individual data points are shown for all bar graphs. *P < 0.05; **P < 0.01; ***P < 0.001; n.s., not significant.

Since ZnT8 is the key element to bridge zinc transportation and cellular stress, we hypothesized inhibiting ZnT8 would inhibit zinc transportation to protect against cellular stress in β cell (**Figure** **3**a). To validate the causative role of ZnT8 mediated zinc transportation, we knocked out ZnT8 in *WFS1^−/−^* hESCs and differentiated them into SC‐β cells (hereinafter referred to as *WFS1^−/−^ZNT8^−/−^*) (Table S3, Supporting Information). Compared to *WFS1^−/−^* SC‐β cells, there was a significant abolishment of zinc transportation and reduction of stress granule formation in the *WFS1^−/−^ZNT8^−/−^* SC‐β cells, as quantified by scoring Zinquin intensity (Figure 3b,c) and G3BP1 intensity (Figure 3d,e).

To determine the changes of β cells when reducing ZnT8, we examined the SC‐β cells from WT, *WFS1^−/−^*, and *WFS1^−/−^ZNT8^−/−^* β cell clusters, respectively. We measured the size of SC‐β cell in each group and found that *WFS1^−/−^ZNT8^−/−^* group showed a significantly increased size, compared to *WFS1^−/−^* counterparts (Figure 3f). Of significance, in *WFS1^−/−^ZNT8^−/−^* group, the percentage of β cells (INS^+^NKX6.1^+^) significantly increased compared to *WFS1^−/−^* SC‐β cells (Figure 3g,h). At the same time, we also examined the cell death events in the *WFS1^−/−^ ZNT8^−/−^* SC‐β cell by Annexin V staining, revealing that cell death rate in the *WFS1^−/−^ZNT8^−/−^* β cell clusters significantly decreased as compared to WT and *WFS1^−/−^* SC‐β cells (Figure 3i,j; Figure S5a,b, Supporting Information). In parallel, overexpression of WFS1 significantly suppressed ZnT8 transcriptional expression and eIF2α phosphorylation (Figure S6a,b, Supporting Information). Since β cell loss caused by *WFS1* deficiency represents a prototype of cellular stress disorder, our results suggested inhibition of ZnT8‐mediated zinc transportation alleviates stress granule formation and β cell loss (Figure 3k). Moreover, our results identified negative regulation of zinc transportation by WFS1 as a potential protective mechanism to preserve human β cell mass against stress‐induced cell death.

Taken together, there are three lines of evidence demonstrate the causal role of elevated zinc transportation for β cell loss in T2D: first, human T2D genetic data supported the causal role of *ZNT8* dysregulation in driving T2D progression. Second, our functional validation data further supported the causal role of zinc transportation in stress granule formation and β cell loss with overexpression of ZnT8 or excessive zinc treatment. Third, genetic lesion like WFS1‐LOF led to upregulated ZnT8 expression, elevated zinc transportation and excessive cell death. When zinc transportation was largely abolished by ZnT8 knockout, there was a significant reduction of stress granule formation and cell death in the *WFS1^−/−^ZNT8^−/−^* SC‐β cells compared to *WFS1^−/−^* SC‐β cells, demonstrating genetic depletion of zinc transportation protects β cell from cellular stress.

### 2.4 A Compound Screening Based on SC‐β Cell Identifies an Inhibitor Targeting ZnT8 Mediated Zinc Transportation

**Figure 4:**
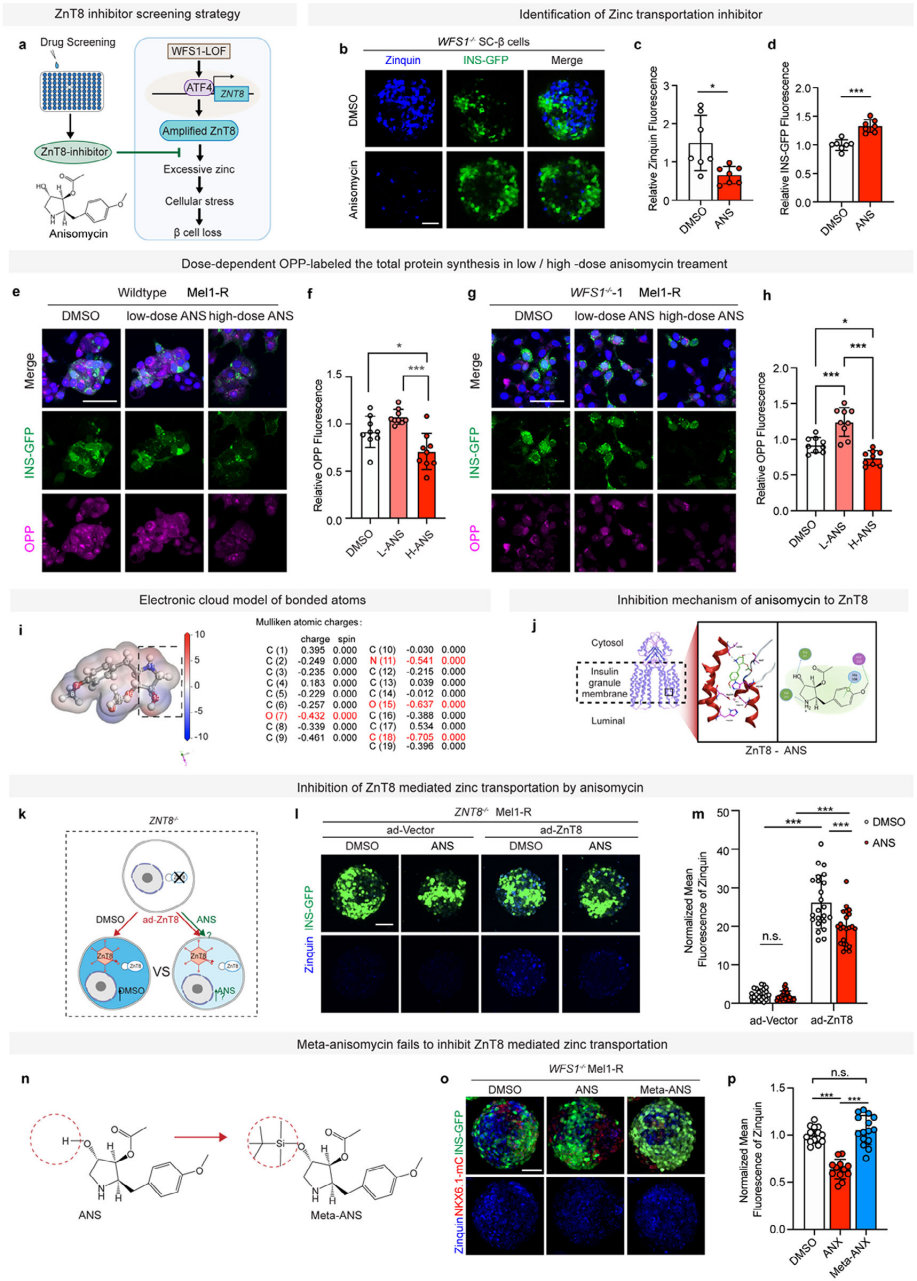
A compound screening based on SC‐β cell identifies an inhibitor targeting ZnT8 mediated zinc transportation. a) Schematic diagram of ZnT8 inhibitor screening strategy based on *WFS1^−/−^* SC‐β cells. b) Representative images of fluorescence of Zinquin (blue) and INS‐GFP (green) in *WFS1^−/−^* ZnT8‐mC SC‐β cells. c,d) Quantification of mean fluorescent intensity of Zinquin (c) and INS‐GFP (d) in b (unpaired two‐tailed *t*‐test, *n* = 7) (normalized with DMSO treated group). e) Representative images of fluorescence of DAPI (blue), INS‐GFP (green), and OPP (magenta) in OPP‐labeled protein synthesis assay of WT SC‐β cells treated with DMSO, low‐dose (25 nm) or high‐dose (1 µm) anisomycin for 48 h. f) Quantification of mean fluorescent intensity of OPP in e (one‐way ANOVA, *n* = 9 of each group) (normalized with DMSO‐treated group). g) Representative images of fluorescence of DAPI (blue), INS‐GFP (green), and OPP (magenta) in OPP‐labeled protein synthesis assay of OPP‐labeled protein synthesis assay of *WFS1^−/−^* SC‐β cells treated with DMSO, low‐dose (25 nm) or high‐dose (1 µm) anisomycin for 48 h. h) Quantification of mean fluorescent intensity of OPP in (g) (one‐way ANOVA, *n* = 9 of each group) (normalized with DMSO‐treated group). i) Electron cloud model based on Mulliken atomic charges. The characters in red represent the atoms involved in the interaction. j) Interactions established after docking the anisomycin to ZnT8 with and without zinc. 2D sketches show the interaction mode between anisomycin and residues. Molecules are green, hydrogen bonds are yellow (3D) or purple (2D). Green lines represent pipi staking and red lines represent pi‐cation. The red helixes and violet sticks together form the active protein site pocket. k) Schematic diagram of overexpressing ZnT8 in *ZNT8^−/−^* SC‐β cells to confirm the inhibition of ZnT8‐mediated zinc transportation by anisomycin. l) Representative images of fluorescence of Zinquin (blue) in *ZNT8^−/−^* SC‐β cells treated with DMSO or 25 nm anisomycin when cultured in the presence of ad‐Vector or ad‐ZnT8. m) Quantification of mean fluorescent intensity of Zinquin in (h) (two‐way ANOVA, *n* = 23 of each group) (normalized with ad‐Vector transduced DMSO treated *ZNT8^−/−^* SC‐β cells). n) Structure of anisomycin and Meta‐anisomycin, red dashed circle indicates shielding group. o) Representative images of fluorescence of Zinquin (blue) in *WFS1^−/−^* SC‐β cells treated by DMSO, anisomycin, and Meta‐anisomycin. p) Quantification of mean fluorescent intensity of Zinquin in (o) (one‐way ANOVA, *n* = 14 (DMSO), 12 (ANS), 15 (Meta‐ANS)). Data are normalized with DMSO‐treated *WFS1^−/−^* SC‐β cells. Scale bars: 50 µm (e,g), 100 µm (b,l,o). Data are mean ± s.d. Individual data points are shown for all bar graphs. *P < 0.05; **P < 0.01; ***P < 0.001; n.s., not significant.

Since abolishment of zinc transportation by genetic ablation of ZnT8 efficiently protects β cells, we hypothesized that a chemical inhibitor of ZnT8 mediated zinc transportation could be of therapeutic efficacy to preserve β cell mass against cellular stress. Thus, we initiated a compound screening targeting ZnT8‐mediated zinc transportation in β cells. Since WFS1‐LOF amplifies ZnT8‐mediated zinc transportation, we designed a compound screening based on *WFS1^−/−^* SC‐β cell labeled by INS (INS‐GFP) and zinc transportation within β cells was visualized by Zinquin (**Figure** **4**a). S7 stage SC‐β cell clusters were seeded into 24 well plates, followed by the administration of compounds from a library containing 1685 chemical inhibitors. After 48 h treatment with the compounds (100 nm), both zinc transportation (Zinquin labeled) and INS expression (INS‐GFP) of *WFS1^−/−^* SC‐β cell clusters were examined under a fluorescence microscope. A compound, anisomycin (ANS), was screened out, which efficiently inhibited zinc transportation but increased the expression of INS in *WFS1^−/−^* SC‐β cell at low‐dose (25 nm) (Figure 4b–d). It should be noted that anisomycin is generally reported as a protein synthesis inhibitor at high‐dose (10 µm),^[^ ^48^ ^]^ hundreds fold higher than that used in our study. And our results revealed that low‐dose (25 nm) anisomycin treatment significantly increases INS‐GFP expression, indicating that it does not inhibit insulin synthesis at low‐dose, suggesting chemical inhibition of zinc transportation by anisomycin at low‐dose is independent of its traditional role as a protein synthesis inhibitor (Figure 4d). To validate that, we performed the O‐propargyl‐puromycin (OPP) labeled nascent polypeptide synthesis assay,^[^ ^16^, ^49^, ^50^, ^51^, ^52^ ^]^ and the total protein synthesis was significantly inhibited in high‐dose (10 µm) anisomycin treated WT or *WFS1^−/−^* SC‐β cells compared to DMSO and low‐dose (25 nm) anisomycin treated group (Figure 4e–h).

In order to analyze interactions between anisomycin and ZnT8, we docked anisomycin to the Cryo‐EM structure of ZnT8 (code: 6XPE, from worldwide protein data bank,^[^ ^53^ ^]^) and calculated electron cloud model with Material Studio. Cluster analysis of anisomycin poses with ZnT8 revealed a strong docking site for interaction which is located in the transmembrane domain of ZnT8.^[^ ^53^ ^]^ There are four residues, Ala87, His106, Asp110, and Val181 in the docking pocket (the electron cloud model shows bonds strength (Figure 4i–j) suggesting that anisomycin's binding to docking pocket in ZnT8. Furthermore, we predicted the minimum free energy of anisomycin binding to the target pocket of ZnT8 based on geometric deep learning provided by MolProphet. To validate the binding site of anisomycin, we predicted the structure of mutagenesis of ZnT8 (Ala87Thr, His106Asn, Asp110His, Val181Ile) with AlphaFold and recalculated the minimum free energy. Consistent with predicted results, fluorescence of Zinquin staining revealed that anisomycin loses the capability of inhibiting zinc transportation of ZnT8 with mutagenesis (Figure S7a–d, Supporting Information).

To further confirm the inhibition of ZnT8‐mediated zinc transportation by low‐dose anisomycin, we overexpressed ZnT8 in *ZNT8^−/−^* SC‐β cells along with low‐dose anisomycin treatment or DMSO, with ad‐Vector as the negative control. ZnT8‐LOF largely abolished zinc transportation, while overexpression of ZnT8 in *ZNT8^−/−^* SC‐β cells dramatically elevated zinc transportation. When treated with low‐dose anisomycin, elevated zinc transportation by ZnT8 overexpression was mainly suppressed compared to DMSO‐treated groups (Figure 4k–m).

At the same time, the results suggested that the nitrogen heterocycle plays an important role in the interaction between anisomycin and ZnT8. Hydrogen bonds are formed between the amine group and Val181, the hydroxyl group and Ala87, as well as the ester bond and Ile86 between the nitrogen heterocycle and ZnT8 (Figure 4j). To further validate the critical role of the nitrogen heterocycle in the inhibition of ZnT8 by anisomycin, we synthesized the Meta‐ANS (Figure 4n; Figure S7e, Supporting Information) to change the electron distribution on the nitrogen heterocycle. As expected, Meta‐ANS lost the capacity to inhibit zinc transportation in *WFS1^−/−^* SC‐β cells in which ZnT8 is upregulated (Figure 4o,p; Figure S7f,g, Supporting Information). Altogether, these results demonstrated that the electron distribution of nitrogen heterocycle of anisomycin is essential for the inhibition of ZnT8‐mediated zinc transportation.

### 2.5 Low‐Dose Anisomycin Treatment Suppresses ISR and Consequent ZnT8 Expression

To further investigate the effect of low‐dose anisomycin treatment on SC‐β cells with *WFS1* deficiency, we performed single‐cell RNA sequencing (scRNA‐Seq) for DMSO or anisomycin treated *WFS1^−/−^* SC‐β cells (Figure S8a, Supporting Information). After quality control and doublet removal, a total of 5277 and 3329 high‐quality single‐cell profiles from *WFS1^−/−^* SC‐β cells treated with anisomycin and DMSO were retained for downstream analysis (Figure S8b, Supporting Information). We identified SC‐β cells as well as other islet cell types from single‐cell transcriptomic profiles (Figure S8c–f, Supporting Information).

**Figure 5:**
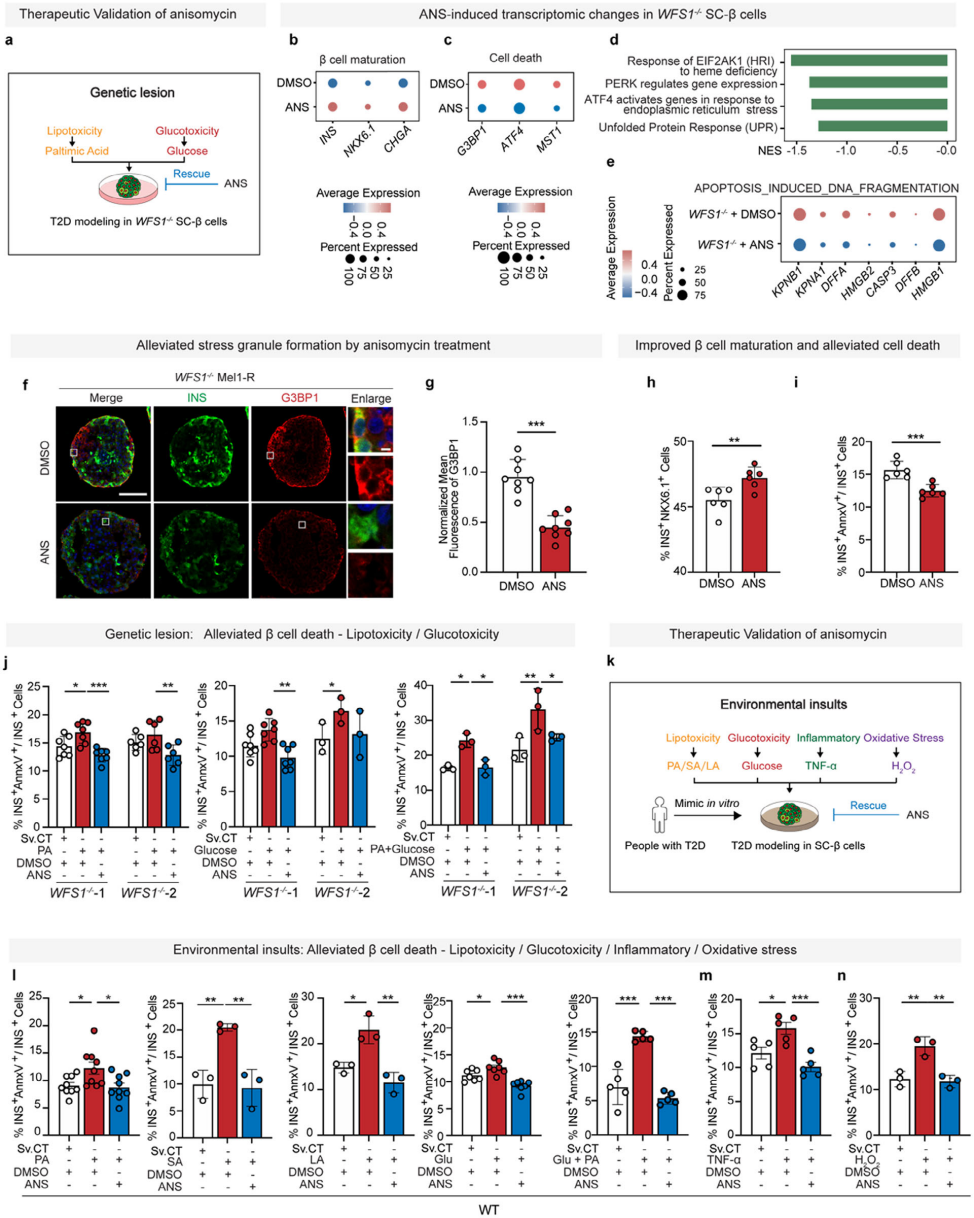
Low‐dose anisomycin protects SC‐β cells from stress‐induced cell death triggered by genetic lesion and environmental insults. a) Schematic diagram of therapeutic validation in *WFS1^−/−^* SC‐β cells. b,c) Dot plot of gene expression levels of markers of β cell maturation and cell death. Dot color shows the average expression, dot size shows the percent of cells without zero expression. d) Reactome pathway enrichment analysis of differentially expressed genes between *WFS1^−/−^* SC‐β cells treated with DMSO and anisomycin for 48 h using GSEA. e) Dot plot of gene expression levels of markers of apoptosis‐induced DNA fragmentation. Dot color shows the average expression, dot size shows the percent of cells without zero expression. f) Representative images of fluorescence of G3BP1 (red), INS (green), and DAPI (blue) in adherent single cell dissociated from *WFS1^−/−^* SC‐β cells treated with DMSO and anisomycin on D10 at the S7 stage for 48 h. g) Quantification of mean fluorescent intensity of G3BP1 in (f) (unpaired two‐tailed *t*‐test, *n* = 8) (normalized with DMSO‐treated group). h) Flow cytometry quantification of *WFS1^−/−^* SC‐β cells on D10 of S7 stage treated by DMSO and anisomycin for 48 h measuring β cell population (INS‐GFP^+^NKX6.1‐mCherry^+^) (unpaired two‐tailed *t‐*tests, *n* = 6). i) Flow cytometry quantification of SC‐β cells on D10 of S7 stage treated by DMSO and anisomycin for 48 h measuring β cell apoptosis (AnnxV^+^INS‐GFP^+^/INS‐GFP^+^) (unpaired two‐tailed *t‐*tests, *n* = 6). j) Flow cytometry quantification of SC‐β cells on D10 of S7 stage treated by DMSO or 25 nm anisomycin when cultured in the presence of vehicle or lipo‐/glucotoxicity conditions (1 mm palmitic acid (PA), 35 mm glucose or both 35 mm glucose and 1 mm PA) for 48 h measuring β cell apoptosis (AnnxV^+^INS‐GFP^+^/INS‐GFP^+^) (two‐way ANOVA, *n* = 7 (*WFS1^−/−^*‐1 in PA group), 6 (*WFS1^−/−^*‐2 in PA group), 7 (*WFS1^−/−^*‐1 in glucose group), 3 (*WFS1^−/−^*‐2 in glucose group), 3 (glucose and PA group)). k) Schematic diagram of therapeutic validation in lipotoxicity (palmitic acid: PA, stearic acid: SA, linoleic acid: LA), glucotoxicity, inflammatory, and oxidative stress. l) Flow cytometry quantification of SC‐β cells on D10 treated by DMSO or 20 nm anisomycin when cultured in the presence of vehicle or lipo‐/glucotoxicity conditions (1 mm PA, 0.6 mm SA, 0.3 mm LA, 35 mm glucose or both 35 mm glucose and 1 mm PA) for 48 h measuring β cell apoptosis (AnnxV^+^INS‐GFP^+^/INS‐GFP^+^) (one‐way ANOVA, *n* = 9 (PA group), 3 (SA group), 3 (LA group), 7 (glucose group), 5 (glucose and PA group)). m) Flow cytometry quantification of SC‐β cells on D10 treated by DMSO or 20 nm anisomycin when cultured in the presence of vehicle or 5 ng mL^−1^ TNF‐α for 48 h measuring β cell apoptosis (AnnxV^+^INS‐GFP^+^ / INS‐GFP^+^) (one‐way ANOVA, *n* = 5). n) Flow cytometry quantification of SC‐β cell on D10 treated by DMSO or 20 nm anisomycin when cultured in the presence of vehicle or 100 µm H_2_O_2_ for 3 h measuring β cell apoptosis (AnnxV^+^INS‐GFP^+^/INS‐GFP^+^) (one‐way ANOVA, *n* = 3). Scale bars: 100 µm (f, left), 5 µm (f, right). Data are mean ± s.d. Individual data points are shown for all bar graphs. *P < 0.05; **P < 0.01; ***P < 0.001; n.s., not significant.

We further identified DEGs between *WFS1^−/−^* SC‐β cells treated with DMSO or anisomycin. We found downregulated genes in *WFS1^−/−^* SC‐β cells treated with anisomycin were enriched in terms associated with SLC transporters (Figure S9a, Supporting Information). For example, *ZNT8* was downregulated in SC‐β cells treated with anisomycin (Figure S9b, Supporting Information). To validate that, we performed qPCR assay on DMSO or anisomycin treated *WFS1^−/−^* SC‐β cells and the result suggested that transcriptional expression level of *ZNT8* was downregulated with anisomycin treatment (Figure S9c, Supporting Information), which was consistent with its reduced protein level (scored by intensity of fused RFP) (Figure S9d–f, Supporting Information). Notably, ATF4 transcriptionally activates ZnT8 expression (Figure 3h–l), and low‐dose anisomycin treatment reduces expression of ATF4 (Figure 5c; Figure S10e, Supporting Information). These results suggested that low‐dose anisomycin treatment reduces expression of ATF4, leading to downregulation of ZnT8 expression. To further validate this, we overexpressed ATF4 with adenovirus and observed ZnT8 expression was upregulated. However, treatment with low‐dose anisomycin in ATF4 O.E. SC‐β cells led to a decrease in ZnT8 expression (Figure S9g,h, Supporting Information). Conversely, downregulating ATF4 expression with shRNA and ISRIB resulted in downregulation of ZnT8 expression (Figure S9i–l, Supporting Information).

**Figure 6:**
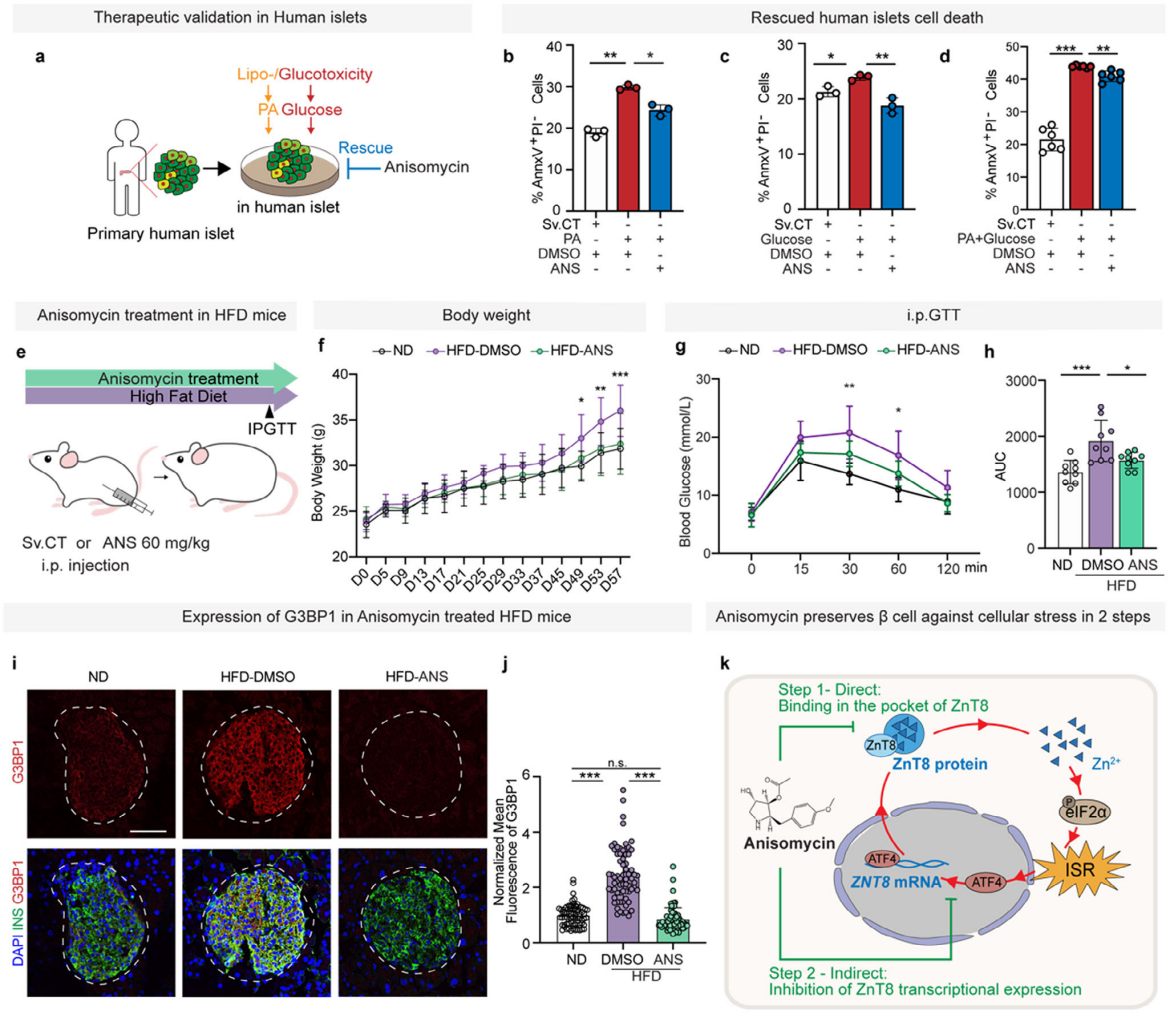
Validation of therapeutic effect of anisomycin against diabetes in primary human islets and diabetic mice. a) Schematic diagram of therapeutic validation in human primary islets. b–d) Flow cytometry quantification of human primary islets treated by DMSO and 20 nm anisomycin when cultured in the presence of vehicle, 1 mm palmitic acid (PA) (b), 35 mm glucose (c) or both 35 mm glucose and 1 mm PA (d), for 48 h measuring β cell apoptosis (AnnxV^+^PI^−^) (one‐way ANOVA, *n* = 3 (PA group), 3 (glucose group), 6 (glucose and PA group)). e) Schematic diagram of therapeutic validation in HFD‐induced diabetic mice, black triangles represent for i.p. glucose tolerance test. f) Bodyweight of ND mice (black circles and line) and HFD‐induced diabetic mice (purple line and dots represent for DMSO treated group, green line and dots represent for 60 mg kg^−1^ anisomycin treated group) (two‐way ANOVA, *n* = 9). g,h) i.p. glucose tolerance test (g, two‐way ANOVA, *n* = 9) and AUC analysis (h, one‐way ANOVA, *n* = 9) of the ND mice (black circles and line) and HFD‐induced diabetic mice (magenta line and dots represent for DMSO treated group, green line and dots represent for 60 mg kg^−1^ anisomycin treated group) on D58. i) Immunofluorescence of pancreas sections of ND mice, DMSO treated and 60 mg kg^−1^ anisomycin treated HFD‐induced diabetic mice with insulin (green), G3BP1 (red), and DAPI (blue). j) Quantification of mean fluorescent intensity of G3BP1 in (i) (one‐way ANOVA, *n* = 70) (normalized with DMSO‐treated ND group). We acquired fluorescence images of representative insulin‐positive islets with a diameter greater than 50 µm, using a fixed exposure time. The expression of G3BP1 was quantified in pancreatic sections from 16‐week‐old male C57BL/6N mice that were fed high‐fat diet and treated with Sv.CT (purple, *n* = 5 mice) or 60 mg kg^−1^ anisomycin (green, *n* = 5 mice) for 8 weeks, alongside age‐matched C57BL/6N mice fed a normal‐diet (white, *n* = 5 mice). A total of 70 islets from each group were examined. The expression level of G3BP1 was assessed based on the mean fluorescence intensity (normalized to the area). k) Working hypothesis of protective mechanism of ZnT8 inhibitor anisomycin against cellular stress in two steps, deduced from the data presented in this study. Scale bars: 100 µm (i). Data are mean ± s.d. Individual data points are shown for all bar graphs. *P < 0.05; **P < 0.01; ***P < 0.001; n.s., not significant.

Taken together, our results demonstrated that low‐dose anisomycin interacts with ZnT8 to inhibit zinc transportation and consequent ISR, indirectly, further leading to downregulation of ATF4‐activated ZnT8 expression to preserve β cell mass against cellular stress in two steps (Figure 6k).

### 2.6 Low‐Dose Anisomycin Protects SC‐β Cells from Cellular Stress‐Induced Cell Death Triggered by Genetic Lesion and Environmental Insults

Next, we recapitulated β cell loss resulting from cellular stress caused by genetic lesions or environmental insults in diabetes to validate the therapeutic effects of anisomycin, the eIF2α phosphorylation induced by O.E ZnT8 was subsequently inhibited (Figure S9m, Supporting Information), and β cell loss induced by O.E ZnT8 was also alleviated by anisomycin treatment (Figure S9n, Supporting Information). Altogether, these results confirmed that anisomycin inhibits ZnT8 mediated zinc transportation to prevent β cell loss. We found that the expression of genes associated with β cell maturation including *INS*, *NKX6.1*, and *CHGA* was upregulated in *WFS1^−/−^* SC‐β cells treated with anisomycin, while genes with cell death including *G3BP1*, *ATF4*, and *MST1* were downregulated (**Figure** **5**a–c). To note, MST1 has been identified as a key regulator for β cell loss.^[^ ^54^, ^55^, ^56^ ^]^ Gene set enrichment analysis (GSEA) suggested that pathways associated with cell stress, such as ATF4 activating genes in response to ER stress and unfolded protein response, were downregulated in SC‐β cells treated with anisomycin (Figure 5c–e). These results proved that anisomycin could rescue β cell function, alleviate cellular stress, and prevent cell death. To note, WFS1‐elicited ISR inhibits total protein synthesis, and our results demonstrated low‐dose anisomycin suppresses ISR, further confirming chemical inhibition of zinc transportation by anisomycin at low‐dose is independent of its traditional role of protein synthesis inhibitor at high‐dose triggering ISR. Since β cell failure caused by *WFS1* deficiency represents a prototype of cellular stress disorder by genetic lesions, we first treated *WFS1^−/−^* SC‐β cells with 25 nm anisomycin for 48 h. In *WFS1^−/−^* SC‐β cells, anisomycin significantly inhibited zinc transportation, eIF2α phosphorylation and stress granule formation (Figure S10a,b, Supporting Information; Figure 5f,g), increased β cell population, and decreased β cell death under both normal conditions and pathological conditions with lipotoxicity or glucotoxicity (Figure 5h–j). Markedly, zinc transportation was elevated by high‐glucose as metabolic stress in *WFS1^−/−^* SC‐β cells, but low‐dose anisomycin significantly inhibited zinc transportation (Figure S10c,d, Supporting Information). Moreover, elevating ER stress caused by *WFS1* deficiency was also decongested by anisomycin treatment, as measured by qPCR of genes related to ER stress, nuclear localization of sXBP1, cytosolic calcium, and mitochondrial depolarization (Figure S10e–k, Supporting Information). On the other hand, we simulated environmental insults with WT SC‐β cells, finding that anisomycin treatment significantly reduced cell death caused by lipotoxicity (1 mm PA, 0.6 mm SA, 0.3 mm LA) and glucotoxicity (35 mm d‐glucose), which were reported to trigger cellular stress in β cells as extrinsic physiological insults.^[^ ^2^, ^32^, ^43^, ^44^ ^]^ Interestingly, anisomycin treatment also dramatically decreased cell death elicited by inflammation (5 ng mL^−1^ TNF‐α) and oxidative stress (100 µm H_2_O_2_) (Figure 5k–n), which are associated with cellular stress and contribute to β cell failure.^[^ ^57^, ^58^ ^]^ Altogether, these results suggested that anisomycin protects SC‐β cells from cellular stress induced by genetic lesion or environment insults.

### 2.7 Validation of Therapeutic Effect of Anisomycin against Diabetes in Primary Human Islets and Diabetic Mice

To further validate the protective effect of anisomycin in primary human islets, we also recapitulated the β cell loss resulting from cellular stress by environmental insults in human islets (**Figure** **6**a). Anisomycin treatment significantly reduced cell death caused by lipotoxicity and glucotoxicity (Figure 6b–d), suggesting that anisomycin could protect primary human islets from environmental insults. To further verify the therapeutic efficacy in vivo, first, we tried different doses in mice by intraperitoneal (i.p.) injection to obtain the proper doses of 40 and 60 mg kg^−1^ for 7 days which significantly decreased zinc transportation in mice islets but did not obviously affect health or survival (Figure S11a–c, Supporting Information). Furthermore, we evaluated efficacy at 40 and 60 mg kg^−1^ in high‐fat diet (HFD)‐induced diabetic mice. Body weight gain was significantly inhibited in both the 40 and 60 mg kg^−1^ group compared to HFD DMSO‐treated control group. However, glucose tolerance was only significantly improved in the 60 mg kg^−1^ group, but not in the 40 mg kg^−1^ group (data not shown), demonstrating 60 mg kg^−1^ is the optimal dose (Figure 6e–h). Remarkably, ZnT8 expression was significantly downregulated, and stress granule formation was largely abolished in islets of anisomycin‐treated HFD mice (Figure 6i,j; Figure S11d,e, Supporting Information). To note, no signs of potential tumorigenesis were detected in the histological images of major organs including heart, liver, spleen, lung, and kidney (Figure S11f, Supporting Information). Taken together, these results validated protective efficacy of anisomycin against diabetes in primary human islets and mice, suggesting a potential novel medication by chemical inhibiting ZnT8 mediated zinc transportation to suppress cellular stress induced cell death to treat diabetes (Figure 6k).

## 3 Discussion

Stress‐induced cell death resulting from chronic ER stress and coupled ISR is considered as a major causative risk for β cell failure in T2D.^[^ ^1^, ^2^, ^8^, ^58^, ^59^ ^]^ Among the three major signaling pathways in ER stress, PERK pathway is activated and induces eIF2α phosphorylation under stress, and it is also interconnected with ISR, consequently limiting mRNA translation, upregulating ATF4, inducing the stress granule formation and cell death.^[^ ^8^, ^11^, ^60^, ^61^ ^]^ Thus, the PERK/eIF2α/ATF4 signaling pathway plays an important role in both ER stress and ISR. On the other hand, metabolic stresses in T2D also debilitate β cells in chronic manners, such as glucotoxicity, lipotoxicity, inflammation, and oxidation.^[^ ^59^ ^]^ Thus, how to resolve cellular stress in β cell failure remains a key question for developing effective medications for T2D. In this study, we discovered the vicious cycle of zinc transportation and cellular stress driving pancreatic β cell loss in diabetes regulated by WFS1–ZnT8–zinc axis. Our results showed that the critical ER stress causative gene, WFS1 deficiency led to significantly increased zinc transportation by upregulating ZnT8 expression, which directly contributed to elevating cellular stress and β cell loss. In turn, heightened cellular stress upregulates ATF4 (cellular stress responsive transcriptional factor), which transcriptionally activates ZnT8 expression with elevated zinc transportation, forming a vicious cycle of zinc transportation and cellular stress. Furthermore, we demonstrated that inhibiting ZnT8‐mediated zinc transportation releases the vicious cycle to protect β cells.

It is important to note that due to the lack of the understanding of the mechanism about how zinc transportation is correlated with cellular stress, no chemical inhibitors targeting ZnT8 have been identified prior to our study. Additionally, most drug discovery relies on animal models or cell lines, which often fail to fully mimic human disease, contributing to a high failure rate (≈90%).^[^ ^22^, ^23^ ^]^ In contrast, based on the study of pathology mechanism, we developed a novel strategy with W*FS1^−/−^* SC‐β cells, to screen inhibitors targeting ZnT8. Moreover, we identified a new therapeutic role for low‐dose anisomycin, traditionally known as a protein synthesis inhibitor. Our findings showed that low‐dose anisomycin directly interacts with ZnT8 to inhibit zinc transportation, alleviating the ISR and disrupting the ATF4–ZnT8 feedback loop. This treatment significantly reduces cell death in SC‐β cells and primary human islets under various stress conditions. In vivo administration of anisomycin in mice protects β cells and prevents high‐fat diet‐induced T2D.

To note, anisomycin was reported as a protein synthesis inhibitor at high‐dose (10 µm).^[^ ^48^ ^]^ However, our results uncovered a novel role of anisomycin at low‐dose (25 nm). Our results demonstrated that low‐dose anisomycin does not inhibit total protein synthesis in SC‐β cell. By contrary, low‐dose anisomycin upregulates the genes’ expressions associated with β cell maturation in SC‐β cells, including *INS* expression. Furthermore, WFS1‐LOF leads to ISR and inhibits total protein synthesis, but low‐dose anisomycin treatment suppresses ISR. Collectively, we demonstrated that low‐dose anisomycin inhibits zinc transportation against cellular stress as a chemical inhibitor of ZnT8, which is different from its traditional role as a protein synthesis inhibitor at high‐dose. Furthermore, anisomycin as a “hit compound” targeting ZnT8 identified through initial screening, requires modifications to improve safety and efficacy before clinical application. While the compound described in this study provides a solid starting point for diabetic protective drug discovery, it remains in the early stages. Our future work will focus on iterative design and optimization to enhance its therapeutic potential, paving the way for eventual clinical applications.

In addition to T2D, genetic loss‐of‐function of *WFS1* also results in β cell loss in diabetes of Wolfram Syndrome (WS), which is a prototype of ER stress disorder. Currently, WS is life‐threatening and there is no effective therapy for WS patients who need exogenous insulin injection and die prematurely around 30 years old.^[^ ^62^ ^]^ The bottleneck lies in poor understanding of the WS pathogenesis in human model. We uncover that both genetic ablation and chemical inhibition of ZnT8 mediated zinc transportation significantly mitigate cell loss in hESCs‐derived *WFS1^−/−^* SC‐β cells, suggestive of a potential therapeutic approach for this severe disease.

Notably, ZnT8 has also been reported as a major autoantigen in T1D,^[^ ^63^ ^]^ and recent advances in investigating T1D revealed that ER stress also triggers T1D.^[^ ^1^, ^2^, ^64^ ^]^ Our study has demonstrated that anisomycin can alleviate ER stress and downregulate ZnT8 expression, suggesting its potential therapeutic application in T1D treatment. Furthermore, pancreatic islet organoids with ZnT8 function inhibition may hold promise for future transplantation‐based therapies for diabetes (both TlD and T2D). However, to fully explore this potential, further studies are required to assess the benefits of disrupting the ATF4–ZnT8 feedback loop in clinical‐grade hESC lines (such as Q‐CTS‐hESC‐1 and Q‐CTS‐hESC‐2).^[^ ^65^ ^]^

## 4 Experimental Section

### Establish an Integrated Human β Cell Atlas—scRNA‐Seq Data

Islet single cell RNA‐seq data were collected from 10 datasets (Table S4, Supporting Information). For datasets with no available gene‐by‐cell count matrix, raw fastq data were downloaded from the SRA database, then mapped to the GRCh38 reference genome by hisat2 and quantified by featureCounts to obtain gene‐by‐cell count matrix.^[^ ^66^, ^67^ ^]^ Seurat was used to cluster cells from each dataset.^[^ ^68^ ^]^ The low‐quality cells were filtered based on the threshold: for the 10× dataset, cells that expressed more than 500 genes and less than 10% of counts aligned to mitochondrial genes, for the Smart‐seq2 dataset, cells that expressed more than 500 genes and less than 20% of counts aligned to mitochondrial genes. Cells that had extremely high number of reads (top 1%) were also excluded. Sctransform was used to perform data normalization and scale, and 30 principal components were used for dimension reduction and Uniform Manifold Approximation and Projection (UMAP) visualization.^[^ ^69^, ^70^ ^]^ Cell cluster was determined by Leiden graph‐clustering method then assigned cell annotation with known marker genes for alpha (*GCG*), beta (*INS*), delta (*SST*), gamma (*PPY*), acinar (*REG1A*), ductal (*CFTR*), stellate (*PDGFRB*), endothelial (*CLEC14A*), and immune cells (*CCL3*). The same procedure was repeated to identify cell types in all datasets, and merged all dataset for integration. The integrated dataset was loaded into Scanpy for normalization and logarithmic transformation.^[^ ^71^ ^]^ Then, 2000 highly variable genes were selected to build a reference scVI model.^[^ ^72^ ^]^ The scVI model was initially trained with a 30‐dimensional latent space and a 0.1 dropout rate across 500 epochs and a batch size of 128. Subsequently, the scANVI model was initialized using default parameters from the trained scVI model. This was done to learn cell representations with the guidance of well‐annotated cell type labels.^[^ ^72^ ^]^ Finally, the integrated human islet atlas, containing overall 388 480 cells, was constructed. This includes 71 178 nondiabetic β cells (from 112 donors) and 30 021 type 2 diabetic β cells (from 48 donors).

### snATAC‐Seq Data

Raw islet snATAC‐seq fastq data were downloaded from the SRA database (Table S4, Supporting Information). The raw fastq files were input in CellRanger atac (V2.1) with the GRCh38 reference genome to generate the fragment file. ArchR was used to preprocess snATAC‐seq data, cells with more than 1000 fragments and TSS enrichment score greater than 3 were only kept.^[^ ^73^ ^]^ 500 bp genome tile matrix was first generated to perform dimension reduction with LSI, then used Harmony to remove the batch effect and visualization with UMAP.^[^ ^69^ ^]^ Cell clusters were determined by Leiden graph‐clustering method then assigned cell annotation with known marker genes expression in gene score matrix (*INS* for β cells, *GCG* for α cells, *SST* for δ cells, *GHRL* for PP/ε cells). After assigning the cell type, MACS2 was used to call β cell‐specific peaks with parameter ‘–nomodel–extsize 150–shift‐75–keep‐dup all ‐q 0.05′.^[^ ^74^ ^]^ The narrow peaks were processed as 500 bp bins for the downstream analysis. Differential peaks between nondiabetic and T2D were calculated with the function getMarkerFeatures in ArchR.

### ChIP‐Seq Data

Raw ChIP‐seq fastq data were downloaded from the SRA database (Table S4, Supporting Information). The raw fastq files were preprocessed by fastp to remove adapters and mapped to the GRCh38 reference genome by bowtie2.^[^ ^75^, ^76^ ^]^ The peak calling was performed by MACS2 with a *q*‐value of 0.01. Summary plots and sample correlation were visualized with deepTools.^[^ ^77^ ^]^ The sample that has a low Pearson correlation coefficient (*r* < 0.8) was removed.

### Hi‐C Data

Raw Hi‐C fastq data were downloaded from the SRA database (Table S4, Supporting Information). The raw fastq files were preprocessed by fastp to remove adapters, then the paired reads were separately mapped to the GRCh38 reference genome by BWA.^[^ ^78^ ^]^ Then the genome with the enzyme cutting site was digested and parsed into genome interaction pairs. At last, the genome interaction pairs were converted to prebinned Hi‐C matrix by Juicer tools.^[^ ^79^ ^]^

### Identify Outlier Donors Using Leave‐One‐Out Cross‐Validation Based on Machine Learning

For dataset with both nondiabetic and type 2 diabetic donors, leave‐one‐out cross‐validation was performed as further quality control. Specifically, for each islet scRNA‐seq dataset, all β cells from one donor were first taken out as testing data and trained a classifier using expression profiles of training β cells from other donors to distinguish β cells from nondiabetic and T2D donors using XGBOOST.^[^ ^80^ ^]^ Then the disease state of testing β cells was predicted from the testing donor using the classifier and calculated the prediction accuracy by comparing predicted disease status and the annotated disease status of these testing β cells. The same procedure was repeated for each donor and prediction accuracy was obtained for all the donors. 32 out of 130 donors exhibited prediction accuracy less than 15%, which indicated these donors did not pass the cross‐validation. Given the heterogeneity of human islet data, donors with high prediction accuracy were retained and 58934 nondiabetic cell (from 68 donors) and 23 250 type 2 diabetic cells (from 30 donors) from 6 datasets were used for further analysis.

### Identify Robust Differential Genes between Nondiabetic and Type 2 Diabetic β Cells

After removing outlier donors from leave‐one‐out cross‐validation, differentially expressed genes between nondiabetic and type 2 diabetic β cells in each dataset were called using MAST (adjusted *p*‐value < 0.05).^[^ ^81^ ^]^ By integrating differential genes from independent cohorts, 31 genes were identified with significant changes in at least 4 datasets (Table S2, Supporting Information).

### Identify Significant Correlation between Genes in β Cell

The β cell pseudobulk count matrix was generated for individual donors. If the data were obtained from 10× technology, donors with more than 50 cells were only retained. For Smart‐seq2 technology, donors with more than 10 cells were retained. Outlier donors identified from leave‐one‐out cross‐validation were not included. Given that single‐nucleus RNA‐seq and single‐cell RNA‐seq data exhibited obvious batch effects, single‐cell RNA‐seq datasets were only used. Then the TPM value of every dataset was calculated and then genes that are present in all datasets were retained. Only the genes with mean TPM > 1 were used for calculating correlations. Overall, 14 876 expressed genes in 90 donors (73 nondiabetic and 17 T2D donors from 9 independent datasets) were obtained. Then the Pearson and Spearman correlations of each TF‐target in 10 shuffles were calculated. In each shuffle, 40% of donors was sampled to calculate the Pearson and Spearman correlation coefficients and the donors with very low gene expression were removed, then a mean value of 10 shuffles is used as the averaged Pearson correlation coefficient and Spearman correlation coefficient. FDRs were then calculated using the Benjamini–Hochberg procedure.^[^ ^82^ ^]^ The genes with averaged correlation coefficient larger than 0.8 with ZnT8 were selected to perform GO enrichment by clusterProfiler.^[^ ^83^ ^]^

### Infer Upstream Regulator of ZnT8 in β Cell from Multiomics Data

snATAC‐seq peaks of β cells were classified as proximal and distal cis‐regulatory elements (CREs) based on the distance between peaks and the transcription start site (TSS). Peaks within and outside +2000/−500 of a TSS were considered proximal and distal, respectively.^[^ ^28^, ^36^ ^]^ For distal CREs, the Activity‐by‐Contact (ABC) model was first applied to compute the ABC Score for each CRE–gene pair as the product of Activity (chromatin accessibility) and Contact (interaction frequency) with the input of islet H3K27ac ChIP‐seq and β cell‐specific scATAC‐seq and Hi‐C data.^[^ ^84^ ^]^ For CREs, R package motifmatchr was used to scan the TF binding sites within each CRE and assign TF‐target relationship with TRANSFAC motif database (2018.3 version).^[^ ^37^ ^]^ ATF4 was identified as one upstream regulator of ZnT8.

### Human Islets Isolation

Human primary islets were isolated from freshly obtained pancreatic tissue, which was collected from donors at the Biobank‐Pancreas Group of the First Affiliated Hospital of Naval Medical University (Approval number: CHEC2018‐111). The isolation protocol was modified from the guidelines of the Clinical Islet Transplantation consortium.^[^ ^85^, ^86^ ^]^ In brief, after removing the peripancreatic fat and connective tissue, the pancreas was perfused at multiple points with 0.5 mg mL^−1^ of Liberase TL (Roche) on ice using a blunt porous injection needle. The perfused pancreas was then cut into 1 cm^3^ pieces and digested in the Ricordi Chamber (BioRep). The digested tissue was separated and purified by continuous density gradient centrifugation based on iodixanol (1.060–1.100 ± 0.01 g mL^−1^). Fractions with ≥85% purity were used for subsequent experiments. The islets were cultured in a specialized medium (CMRL1066 supplemented with 2% human albumin, 1 mg mL^−1^ nicotinamide, and 0.6% penicillin/streptomycin).

### Stem Cell Culture

Two stem cell lines were used in SC‐β cell differentiation protocols. Mel1‐Reporter line (*INS^w/GFP^ NKX6.1:2a:mCherry*) was a gift from Prof. Xin Chen (Shanghai Institute of Biochemistry and Cell Biology, Shanghai, China). HuES8 line was a gift from Prof. Qiurong Ding (Shanghai Institute of Nutrition and Health, Shanghai, China). P3 Swiss Webster MEFs were homemade and Swiss Webster mice were gifts from Prof. Xin Chen.

One day before stem cell passaged, 3.5 cm plates were coated by 1:6 diluted Matrigel (Corning, #354277) and following seeded with P3 MEFs (2 × 10^5^ cells per well) with HES medium. Stem cells were passaged every 3–4 days with TrypLE (Gibco, #12604013) at 1:6 to 1:8 split dilutions, and grown on Matrigel‐MEF coated plates with HES medium at 37 °C with 5% CO_2_.

HES medium: DMEM/F12 (Gibco, #11330‐021) supplemented with 20% Knockout Serum Replacement (Gibco, #A3181502), 1 × NEAA (Gibco, #11140050), 1 × l‐glutamine (Gibco, #25030081), 0.05 mm β‐mercaptoethanol (Invitrogen, #21985023), and 10 ng mL^−1^ bFGF (R&D system, #233‐FB/CF).

### Generation and Identification of Isogeneic Mutant Human Embryonic Cell Lines—*WFS1^−/−^* and *ZNT8^−/−^* hESC Line

One or two sgRNAs targeting two different loci on target gene were cloned into P2U6 vectors (gift from Prof. Zhili Rong, Southern Medical University, Guangzhou, China). Mel1‐Reporter line and HuES8 were transfected with P2U6 vectors carrying Cas9 and one or two sgRNAs. After 24–48 h generation, 400 ng mL^−1^ puromycin was added into HES medium for selection, and HES medium with puromycin was replaced after 2 days selection. Single‐cell clones were picked and generated 5–7 days later; PCR was used to validate the knockout efficiency.

### Mel1 ZnT8 Reporter Line

Two sgRNAs targeting two different loci upstream 5′ UTR on *ZNT8* were cloned into P2U6 vectors, and sequences of exon 8 of *ZNT8* were cloned into Pc.DNA3.1 vectors, and the stop coden was replaced by the sequence of mCherry or RFP with stop code at the end. The Mel1 (*INS^w/GFP^*) line was transfected with the two plasmids (with the ratio of 1:2), and 400 ng mL^−1^ puromycin was added into HES medium for selection after 24–48 h generation. Single‐cell clones were picked and generated 5–7 days later. PCR was used to validate the knockout efficiency. All primers used were listed in Table S5 of the Supporting Information.

### SC‐β Cell Differentiation

For initiation of SC‐β cell differentiation, Mel1‐Reporter cells (2 × 10^6^ cells mL^−1^) and HuES8 (1.5 × 10^6^ cells mL^−1^) cells were seeded at in HES medium supplemented with 5 µm Y27632. The differentiation was started 48 h later when cell got 80–90% confluency by changing medium to Day 0 as described in Table S6 of the Supporting Information.

### Quantitative RT‐PCR Analysis

Total RNA was isolated using TRNzol Universal (Tiangen, #DP424) according to instructions by the manufacturer. 0.5 µg of total RNA was used to generate first strand cDNA using the FastQuat RT Kit (Tiangen, #KR106‐02). First strand cDNA products were diluted 18‐fold and used as qPCR templates. SYBR Green‐based qPCR (Tiangen, #FP20502) was carried out using the Roche 96 Lightcycler. Triplicate reactions were carried out for each sample. GAPDH was used as a control to normalize target gene expression. All primers used were listed in Table S7 of the Supporting Information.

### ChIP‐qPCR Analysis

The SC‐β cells were digested and resuspended by cold DPBS to 10^6^ cells mL^−1^, then 37% formaldehyde solution was added to cell suspension to 1% final concentration followed by 10 min rotation in RT. Then 125 mm glycine was added to finish cross‐link followed by 5 min rotation in RT. After centrifuge at 2000 rpm for 5 min at 4 °C, supernatant was discarded and cell pellet can be stocked in −80 °C for several weeks. 10 µL Dynabeads protein A was put into 1.5 mL^−1^ Eppendorf tubes on magnet, and washed three times with RIPA buffer. 1 µg ATF4 antibody was added with 200 µL RIPA buffer into beads and rotated at 4 °C for 3 h. Lysis buffer was added into cell pellet up to 130 µL, mix and vortex thoroughly on ice. Cell pellet was sonicated (Peak power: 75, Duty Factor: 20, Cycle: 200) and transferred to new tube for centrifuge for 20 min at full speed at 4 °C. After all supernatant removed, sample was diluted to ≈600 µL with RIPA buffer, 1/20 volume of sample was kept as input sample, and 100–200 µL sample was added into beads and rotated overnight at 4 °C.

The second day, the sample was put on the magnet and washed with RIPA buffer twice and TE buffer once (rotate for 5 min each time), 200 µL complete elution buffer with protease K was added into each sample followed by shaking for 2 h at 68 °C at 1350 rpm. After reverse‐crosslink, the sample was put on the magnet and supernatant was transferred to another new tube, then 200 µL of phenol: chloroform, 1/5 volume NaAc, and 1 µL glycogen, the same volume of precold isopropanol was added into sample and input in sequence and mixed thoroughly. The mixture was precipitated in −80 °C for 30 min, then centrifuged at full speed for 20 min at 4 °C and washed with 70% ethanol twice. DNA pellet was resuspended with 18 µL ddH_2_O, and qPCR was performed after concentration test. Primers targeting *ZNT8* were listed in Table S7 of the Supporting Information, and ATF4 antibody was listed in Table S8 of the Supporting Information.

### Western Blot

The SC‐β cells were washed with cold DPBS and harvested into cold RIPA lysis buffer (20 mm Tris‐HCl, 150 mm NaCl, 1% Triton, 500 mm Na_3_VO_4_, 5 mm NaF, added with 1% protease inhibitor cocktail), and the bicinchoninic acid protein quantification kit (YEASEN, #20201ES76) was used to measure protein concentrations to get equal amounts of proteins. The lysates were mixed with loading buffer and boiled for 5 min, then centrifuged at 12 000 g in precooled centrifuge for 15 min. The prepared lysates were loaded onto 10% SDS/PAGE gels (Epizyme, #PG112), resolved by electrophoresis, and proteins were transferred to nitrocellulose membranes (PALL, #66485). The nitrocellulose membranes were blocked in a solution of PBST (DPBS supplemented with 0.1% Tween‐20) containing 5% nonfat milk for 1 h, followed by incubation with primary antibody at 4 °C overnight. The second day, after being washed three times with TBST, the membranes were incubated in the secondary antibodies for 1 h. The blots were visualized by enhanced chemiluminescence and exposed to Amersham Imager 600 or Tanon 4600. All immunoblotted antibodies were listed in Table S8 of the Supporting Information.

### Immunocytochemistry Analysis

For adherent cells, cells were washed once with DPBS and fixed with 4% PFA for 30 min at room temperature (RT) and blocked in a solution of DPBS containing 5% normal donkey serum (Jackson Lab) and 0.1% TritonX‐100 for 30 min at room temperature, followed by incubation with primary antibody at 4 °C overnight.

For 3D cultured spheroids, spheroids were washed once with DPBS and fixed with 4% PFA for 30 min at RT. After being washed three times, spheroids were embedded and frozen in OCT (SAKURA) and sectioned with Leica CM1950. Sections were blocked in a solution of PBST (DPBS supplemented with 0.1% TritonX‐100) containing 5% normal donkey serum (Jackson Lab) for 30 min at room temperature, followed by incubation with primary antibody at 4 °C overnight.

After washing three times, sections were incubated with secondary antibodies for 30 min at room temperature. Alexa fluor secondary antibodies were obtained from Invitrogen (1:500). The images were quantified using ImageJ. All antibodies used were listed in Table S8 of the Supporting Information.

### Zinc Imaging

For SC‐β cells or monolayer cells staining, spheroids or cells were washed once with prewarmed DPBS, then stained with 20 nm Zinquin (Sigma, #Z2251) in FACS buffer (DPBS with 0.5% BSA) in the dark at 37 °C with 5% CO_2_ for 30 min as previously performed.^[^ ^87^ ^]^ The spheroids or cells were washed once with FACS buffer and observed by Zeiss LSM880, Leica SP8 or Olympus FV3000 microscope immediately, and Zinquin fluorescence intensity was measured by ImageJ.

For mouse islet staining to test the optimal dose of anisomycin, 8‐week‐old male C57BL/6N mice (*n* > 3) were treated with Sv.CT and anisomycin (10, 40, 60, and 100 mg kg^−1^) via i.p. injection every 48 h. On day 7, mice were sacrificed, then islets were isolated and stained with 20 nm Zinquin, following the same procedure used for SC‐β cells. The stained islets were observed by Zeiss AXIO IMAGER M2 immediately, and Zinquin fluorescence intensity was measured by ImageJ.

For frozen section staining, sections were stained with 30 µm TSQ^[^ ^88^ ^]^ (Sigma, # SML2656) in the dark with secondary antibody. The images were quantified using ImageJ.

### Calcium Imaging

Fluo‐4‐AM dye powder (Thermo, #F14201) was dissolved to 0.5 mm with DMSO supplemented with 0.02% pluronic acid F‐127 and stored in dark.

Spheroids or cells stained with fresh S7 medium with 2.5 µm Fluo‐4‐AM in the dark at 37 °C with 5% CO_2_ for 60 min. The spheroids or cells were washed once with prewarmed FACS buffer and observed by Zeiss LSM880 or Leica SP8 microscope immediately, and Fluo‐4‐AM fluorescence intensity was measured by ImageJ.

### Live‐Cell Imaging of Mitochondria and Intracellular Zinc Distribution

To detect intracellular distribution of free Zn^2+^, cells were loaded with 20 nm Zinquin in HBSS. After washing with FACS buffer, cells were treated (30 min, 37 °C) with 100 nm MitoTracker Red CMXRos (Beyotime, C1035) to stain the mitochondria. Cells were washed with FACS buffer and observed by Leica SP8 or Olympus FV3000 microscope immediately, and fluorescence intensity were measured by ImageJ.

### Mitochondrial Fragmentation

Following the desired treatments, cells were treated with 100 nm MitoTracker Red CMXRos (30 min, 37 °C) in FACS buffer. After washing with FACS buffer, cells were observed by Olympus FV3000 microscope immediately.

### Mitochondrial Depolarization Test

The HuES8 SC‐β cells were dissociated into single cells by 0.25% trypsin and seeded onto the 3.5 cm Matrigel‐coated glass bottom petri dish. After treatment, the cells were loaded with 5,5′,6,6′‐tetrachloro‐1,1′,3,3′‐tetraethylbenzimidazolyl‐carbocyanineiodide (Invitrogen, #65‐085138) at 37 °C for 30 min. The stained cells were then examined by Leica SP8 or Olympus FV3000 microscope. The mitochondrial membrane was visible either as green (488 nm) for monomers or red (530 nm) for aggregates, and fluorescence intensity was measured by ImageJ.

### Annexin V Cellular Apoptosis Analysis

For SC‐β cell staining, spheroids were washed once with prewarmed DPBS and stained with Annexin V‐Alexa Fluor 647 apoptosis detection kit (Yeasen, #40304ES60) in the dark. The spheroids staining was taken Z‐Stack 3D images by Zeiss LSM880 microscope, and Annexin V fluorescence intensity was measured by ImageJ.

For FACS assay, cells were dissociated by 0.25% trypsin‐EDTA and washed once with cold DPBS and then stained with Annexin V‐Alexa Fluor 647 in the dark, then cells were analyzed within 30 min by BD FACS Canto II.

### Drug Screening

To perform the drug screen, *WFS1^−/−^* Mel‐1 ZnT8 reporter line (INS‐GFP ZnT8::mCherry) was generated and differentiated to SC‐β cells, *WFS1^−/−^* Mel‐1 ZnT8::mC‐derived SC‐β cells were treated with compounds from library (Selleck, #L1100), with one compound per well at 100 nm. DMSO treatment was used as a negative control. After 48 h incubation, cells were stained with Zinquin and analyzed using Zeiss AXIO IMAGER M2 immediately. Compounds decreasing the fluorescence level of SC‐β cells were picked as primary hits.

### Dock and Electron Cloud Calculation

Cryo‐EM structure with code 6XPE had been downloaded from the Worldwide Protein Data Bank. 6XPE with Prime was first processed, steps were as follows: assign bond orders, add hydrogens, create zero‐order bonds to metals, create disulfide bonds, fill in missing side chains and missing loops, generate het states, optimize hydrogen bond, remove waters and restrained minimization. Meanwhile, energy of ANS and Meta‐ANS with OPLS4 force field was minimized and possible states at target pH: 7.4 ± 2.0 were generated. Sitemap module was applied to predict potential binding pockets with processed 6XPE. For screening protocol, glide was employed to dock ANS and Meta‐ANS against 6XPE, picked up top five candidate binding sites ranked by site‐point and used the default parameters to generate 100 poses for each site under XP precision in case of zinc and none zinc. After cluster analysis, representative pose was selected to calculate electron cloud model based on Mulliken atomic charges in Material Studio.

### Mouse Studies

C57BL/6N mice were housed in the animal facility of Tongji University, Shanghai, China. All experiments were conducted in compliance with the Guide for the Care and Use of Laboratory Animals and approved by the Biological Research Ethics Committee of Shanghai East Hospital, Tongji University (TJAB03624101).

For studies on β cell protection in HFD‐induced diabetic C57BL/6N mice, 8‐week‐old male mice were treated with anisomycin via i.p. injection (60 mg kg^−1^ every 48 h) while being fed an HFD. Control groups included mice on a normal diet and HFD‐fed mice treated with DMSO. On day 58 of anisomycin treatment, an i.p. glucose tolerance test (i.p. GTT) was performed by fasting the animals for 16 h. Blood glucose levels were measured at 0, 15, 30, 60, and 120 min following an intraperitoneal injection of glucose (2 g kg^−1^ body weight).

### Mouse Islet Isolation and Purification

Mouse pancreas was perfused by 2–3 mL digestion solution (low‐glucose DMEM medium containing 1% Pen/Strepand, 25 mm HEPES, 0.5 mg mL^−1^ collagenase P) with a 27 gauge bended needle from the bile duct. Every 2–3 dissected pancreases were placed in a 50 mL conical tube on ice, and then incubated at 37 °C for 12–15 min followed by hand shaking for 10–15 s after adding 20 mL quenching buffer (low‐glucose DMEM medium containing 10% FBS, 1% Pen/Strep, and 25 mm HEPES). Then the conical tubes were centrifuged at 960 rpm for 2 min at 10 °C. The supernatant was poured off and repeated the process for 2–3 times. The tissue was resuspended in 20–30 mL quenching buffer, filtered through a 250 µm wire mesh, centrifuged at 980 rpm for 2 min and keep the pallet was retained.

The pellet was resuspended in 11 mL purificational buffer (5 mL Histopaque 1077 and 6 mL Histopaque 1199) then slowly added 11 mL of digestion solution. The tissue was centrifuged for 20 min at 1700 rpm with very slow acceleration and no braking. The islet layer was collected and filtered through a 70 µm strainer, then hand‐picked under the microscope.

### Single‐Cell RNA‐Seq Data Analysis

The scRNA‐seq libraries were generated with the DNBelab C Series Single‐Cell Library PrepKit (MGI, #940‐000047‐00), and qualification was performed using Qubit ssDNA Assay Kit (Q10212) and Agilent Bioanalyzer 2100. All libraries were further sequenced by the MGISEQ‐2000RS with pair‐end sequencing. Raw sequencing data were aligned to GRCh38 human reference genome. The transcriptional object was generated from Seurat. Low quality cells were removed with less than 200 or more than 9000 detected genes, or mitochondria gene content more than 10%. Genes were filtered with less than three cells detected in each sample. R package DoubletFinder was used to remove doublets.

SCTransform‐v2 was used for normalization. IntegrateData functions were used to perform integration. RunPCA function in Seurat was used for principal component analysis. RunHarmony function was used for removing batch effects. 40 components were selected for cell clustering and downstream analysis. FindNeighbors and FindClusters (the resolution was adjusted to 1.0) function were run to get 16 clusters. Dimensionality reduction was performed by RunUMAP function in Seurat.

Marker genes were identified of each cluster to define cell types using FindAllMarkers in Seurat. According to known gene markers including *GCG*, *INS*, *SST*, *CHGA*, *GHRL*, *FEV*, those different clusters were defined. Finally, 16 clusters were merged to 7 cell types including α cells (*GCG*), β cells (*INS*), δ cells (*SST*), ɛ cells (*GHRL*), pancreatic progenitor cells (*SOX9)*, EC cells (*FEV*), and proliferation cells (*MKI67*). Particularly, EC cells were a type of cells that both express serotonin synthesis and enterochromaffin markers such as *FEV*,^[^ ^89^ ^]^ and were observed in previous reports on in vitro pancreatic differentiation from pluripotent stem cells.^[^ ^89^, ^90^ ^]^

The differential expression gene levels in β cells were compared between two conditions. DEGs were identified using FindMarkers function in Seurat. Pathway analysis was based on REACTOME pathway database using R package clusterProfiler.

### Statistical Analysis

 *n* = 3 independent biological replicates if not otherwise specifically indicated. Data management and calculation were performed using GraphPad Prism 8. Comparisons between two groups were done using the paired or unpaired two tailed Student's *t*‐test. Multiple comparisons were done using one‐way ANOVA or two‐way repeated measures ANOVA.

n.s. indicates nonsignificant difference. *p* values < 0.05 were considered statistically significant, and the following notations were used in all figures: *p < 0.05, **p < 0.01, and ***p < 0.001. All error bars shown represent SD.

## Conflict of Interest

The authors declare no conflict of interest.

## Author Contributions

R.H., Q.M., and Y.K. contributed equally to this work. R.H., Q.M., R.Z., and W.L. conceived, designed the experiments, and interpreted the results with M.G. R.H. and Z.W. performed the experiments with assistance from Q.M., X.W., M.X., Y.S., T.X., Q.H., X.W., and X.L. Y.K. and G.W. performed the integrated human β cell atlas, machine learning, and downstream analysis. S.C., W.X., X.W., M.X., and S.C. performed chemoinformatics analysis and constructed Meta‐ANS. Z.W., T.X., and Q.H. performed compound screening. X.C. and Y.Y. performed bioinformatics analysis. M.G. and Y.L. performed the human islets isolation and collected the human pancreas samples. M.G. and R.Z. provided critical suggestions to the overall study. R.H., Q.M., Z.W., M.G., G.W., and W.L. wrote the manuscript.

## Supporting information



Supporting Information

## Data Availability

The raw and processed data used for this study have been deposited on GEO with accession number GSE249735. Custom codes for the main analysis used in this study have been deposited on GitHub at https://github.com/yuanlizhanshi/Islet_ML and https://github.com/xychen24/Code_for_Inh19.
