# Supplementary material for: A Compound Screen Based on Isogenic hESC‐Derived β Cell Reveals an Inhibitor Targeting ZnT8‐Mediated Zinc Transportation to Protect Pancreatic β Cell from Stress‐Induced Cell Death
Source: Adv Sci (Weinh). 2025 Apr 7;12(20):2413161. doi: 10.1002/advs.202413161 (PMC12120731; doi:10.1002/advs.202413161)
Supplement: Supplementary file 1 — Supporting Information [file ADVS-12-2413161-s001.pdf]

ADVANCED SCIENCE
Open Access

Supporting Information

for *Adv. Sci.*, DOI 10.1002/advs.202413161

A Compound Screen Based on Isogenic hESC-Derived β Cell Reveals an Inhibitor Targeting ZnT8-Mediated Zinc Transportation to Protect Pancreatic β Cell from Stress-Induced Cell Death

Rui Hu, Qing Ma, Yunhui Kong, Zhaoyue Wang, Minglu Xu, Xiangyi Chen, Yajuan Su, Tinghui Xiao, Qing He, Xuan Wang, Wenjun Xu, Yiling Yang, Xushu Wang, Xiaobo Li, Yanfang Liu, Shuangshuang Chen, Rui Zhao, Meng Guo, Gaowei Wang* and Weida Li**

Table S1 _ Wang et al.

Donor	Sample ID	Diabetes status	ID	Human islet resource center	Islet index	Age	Sex	Ethnicity	BMI	HbA1c	Purity _%	Cause of death	Medical record	Duration of T2D	Treatment history
JYH792	SAMP-692	Non-diabetic	A0018	City of Hope National Medical Center	1.3	37	M	White/ Caucasian	29.5	5.5	0.75	CVA	Tox rpt benzodiazepine and cannabinoids, benzo may have been prescription due to shoulder injury	NA	oral medication
JYH809	SAMP-689	T2D	A0016	City of Hope National Medical Center	1.4	57	M	Hispanic/Latino	23.3	7.3	0.73	CVA (stroke)	T2D	0-5 year	meds 10 years
MM108	SAMP-1613	Pre-T2D	A0011	City of Hope National Medical Center	1.3	34	F	White/ Caucasian	31.5	5.7	0.8	Anoxia	NA	NA	NA
MM109	SAMP-1608	T2D	C0019	City of Hope National Medical Center	1.34	54	M	Hispanic/Latino	23.6	5.8	0.83	Cerebrovascular/ stroke	T2D,HTN, cholesterol,,gastrointestinal disease, heart valve was replaced	0-5 year	oral medication, insulin independent
MM110	SAMP-1695	Non-diabetic	C0026	City of Hope National Medical Center	1.32	20	M	Hispanic/Latino	35.8	4.6	0.95	Head trauma	NA	NA	NA

MM12	SAMP-1097	T2D	A0034	City of Hope National Medical Center	1.01	52	M	Hispanic/Latino	29.9	8.3	0.75	COD CVA	T2D HTN and HChol 8y	9 year	metformin and insulin non-compliant non-compliant
MM120	SAMP-1611	T2D	A0026	City of Hope National Medical Center	1.61	36	F	Hispanic/Latino	44.3	6.9	0.8	Anoxia/DCD	T2D HTN Hchol, inhaler for anxiety,	7 years	no meds
MM121	SAMP-1607	T2D	B0004	University of Pennsylvania	1.27	54	M	Black/African American	33.7	8	0.9	Cerebrovascular/stroke	T2D, HTN, CVA	0-5 year	diet, oral medication, insulin independent
MM122	SAMP-1605	T2D	C0024	Scharp-Lacy Research Institute	0.84	66	F	Hispanic/Latino	29.1	7.2	0.85	Cerebrovascular/stroke	T2D	6-10 years	oral medication, insulin independent
MM123	SAMP-1696	Non-diabetic	C0027	University of Pennsylvania	1.7	24	M	White/Caucasian	23.9	5	0.95	Anoxia	NA	NA	NA
MM124	SAMP-1697	Non-diabetic	C0028	City of Hope National Medical Center	1.36	45	M	Hispanic/Latino	32.9	5.4	0.85	Cerebrovascular/stroke	Hypertension 6 yrs took meds unknown	NA	NA
MM51	SAMP-1150	T2D	C0021	Scharp-Lacy Research Institute	0.8	66	F	White/Caucasian	29.8	6.5	0.95	Cerebrovascular/stroke	T2D, Hypertension	0-5 year	diet, oral medication, insulin independent

MM54	SAMP-1144	T2D	A0020	City of Hope National Medical Center	1	51	F	Hispanic/Latino	43.3	9.6	0.85	Anoxia	HTN, T2D HLD, hypothyroidism, iron deficiency anemia, obesity	NA	oral medication
MM55	SAMP-1138	Pre-T2D	A0028	City of Hope National Medical Center	1.46	64	M	White/Caucasian	34.6	5.8	0.8	Head trauma	HTN , hyperthyroidism,	NA	NA
MM56	SAMP-1136	Non-diabetic	A0032	City of Hope National Medical Center	1.63	45	M	Hispanic/Latino	26.5	4.6	0.8	CVA	Previous CVA, on blood thinners, Hchol 5y meds, heavy alcohol use;	NA	NA
MM57	SAMP-1149	T2D	C0017	Scharp-Lacy Research Institute	1	45	M	White/Caucasian	27.2	6.5	0.85	Cerebrovascular/stroke	T2D	0-5 year	diet, oral medication, insulin independent
MM59	SAMP-1134	Non-diabetic	A0019	City of Hope National Medical Center	1.02	61	M	White/Caucasian	27	5.6	0.8	Head trauma/GSW	HTN (atorvastatin), anxiety (Xanax), daily marijuana use (Rx), tox pos THC, benzodiazepines, and amphetamines	NA	NA

MM60	SAMP-1145	T2D	A0024	City of Hope National Medical Center	1.3	59	M	White/ Caucasian	36.7	6.8	0.83	DCD after CVA	T2D,HTN and cardiac arrhythmia meds, previous CVA, alcohol abuse	NA	NA
MM61	SAMP-1139	Pre-T2D	A0029	City of Hope National Medical Center	1.14	63	F	Hispanic/Latino	28.22	5.7	0.8	Anoxia following CVA	NA	NA	NA
MM77	SAMP-1141	Pre-T2D	C0013	Scharp-Lacy Research Institute	0.75	57	F	White/ Caucasian	27.5	5.9	0.85	Anoxia	NA	NA	NA
MM78	SAMP-1142	Pre-T2D	C0014	Scharp-Lacy Research Institute	1.05	33	M	Black/ African American	30.8	5.7	0.9	Head trauma	NA	NA	NA
MM79	SAMP-1148	T2D	C0015	Scharp-Lacy Research Institute	0.76	26	M	Black/ African American	28.7	6.5	0.9	Anoxia	T2D	0-5 year	NA
MM80	SAMP-1137	Non-diabetic	C0016	Scharp-Lacy Research Institute	1.1	48	M	Hispanic/Latino	32.4	5.6	0.95	Cerebrovascular/ stroke	amphetamines	NA	NA
MM81	SAMP-1151	T2D	A0022	City of Hope National Medical Center	1.27	53	M	Black/ African American	31.2	7.4	0.8	CVA	T2D HTN, HLD, COPD, cigarettes, moderate alcohol, 2016 triple bypass and pig heart valve;	25 years	non compliant

MM86	SAMP-1602	Non-diabetic	A0023	City of Hope National Medical Center	1.87	46	M	White/ Caucasian	34.4	5.6	0.85	Head trauma/MVA	OTC allergy meds,	NA	NA
MM87	SAMP-1500	Pre-T2D	A0030	City of Hope National Medical Center	0.93	49	M	Hispanic/Latino	34.1	6.1	0.8	DCD following CVA	HTN, cannabis 1-2/wk, cigarettes,	NA	NA
MM88	SAMP-1499	T2D	A0031	City of Hope National Medical Center	1.41	45	M	White/ Caucasian	36.4	6.6	0.73	CVA/stroke	Undiagnosed T2D, HTN 4y untreated, alcohol abuse, amphetamines,	NA	NA
MM89	SAMP-1501	Non-diabetic	A0033	City of Hope National Medical Center	1.14	45	F	White/ Caucasian	38	5.3	0.8	COD CVA	HTN >10y	NA	NA
MM93	SAMP-1609	Pre-T2D	A0021	City of Hope National Medical Center	1	61	F	Black/ African American	26.6	5.7	0.98	Anoxia	asthma and depression, tox report cannibinoids, cocaine, amphetamine, heavy alcohol use	NA	NA
MM94	SAMP-1610	T2D	A0025	City of Hope National Medical Center	0.78	35	M	Hispanic/Latino	32.8	10	0.8	Head trauma/MVA	T2D, HTN 4y no meds	NA	NA

MM95	SAMP-1612	Non-diabetic	A0027	City of Hope National Medical Center	1.26	59	M	White/ Caucasian	27.2	5.1	0.9	CVA	HTN, allopurinol for gout, hydrocodone and gabapentin for leg pain;	NA	meds
MM96	SAMP-1498	Pre-T2D	C0022	Scharp-Lacy Research Institute	0.9	50	M	Hispanic/Latino	32.8	6	0.9	Anoxia	MARIJUANA	NA	NA
MM97	SAMP-1503	T2D	C0023	Scharp-Lacy Research Institute	0.91	58	M	White/ Caucasian	32.5	6.7	0.85	Cerebrovascular/ stroke	T2D	0-5 year	diet, oral medication, insulin independent
MM98	SAMP-1606	Non-diabetic	C0025	University of Wisconsin	1.33	51	M	White/ Caucasian	32.8	4.9	0.95	Cardiac arrest s/p choking	HTN, GERD, anemia, anxiety, ex smoker, CHF vs COPD	NA	NA

Table S1 _ Camunas-Soler et al.

donor	status	Sex	Age	BMI	HbA1c
sample3_AGAL381_T2D	T2D	Female	52	21.9	7
sample3_AGJU173_T2D	T2D	Female	52	29.2	9.9
sample3_R230_ND	ND	Male	58	29.412	6.2
sample3_R233_ND	ND	Female	76	19.274	6
sample3_R234_ND	ND	Female	50	31.729	5.7
sample3_R235_ND	ND	Female	53	24.465	5.7
sample3_R237_ND	ND	Male	61	19.676	5.9
sample3_R239_ND	ND	Female	24	22	5.5
sample3_R242_ND	ND	Male	46	20.109	5.9
sample3_R243_ND	ND	Male	39	27.143	5.8
sample3_R247_ND	ND	Male	72	23.9	
sample3_R252_ND	ND	Female	26	25.403	5
sample3_R253_ND	ND	Male	57	25.597	5
sample3_R256_ND	ND	Male	23	32.5	5.4
sample3_R260_ND	ND	Female	73	26.9	6.2
sample3_R264_ND	ND	Male	44	33.771	5.7
sample3_R347_T2D	T2D	Male	57	27.9	6.3

Table S1 _ Xin et al.

donor	status	gender	age
sample16_Non_T2D_4_ND	ND	F	56
sample16_T2D_6_T2D	T2D	M	51
sample16_Non_T2D_8_ND	ND	M	60
sample16_T2D_4_T2D	T2D	F	41
sample16_Non_T2D_7_ND	ND	M	29
sample16_Non_T2D_5_ND	ND	M	27
sample16_Non_T2D_6_ND	ND	M	68
sample16_Non_T2D_9_ND	ND	F	24
sample16_Non_T2D_2_ND	ND	F	32
sample16_Non_T2D_10_ND	ND	M	43
sample16_Non_T2D_1_ND	ND	M	23
sample16_Non_T2D_3_ND	ND	F	23
sample16_Non_T2D_11_ND	ND	F	31
sample16_T2D_5_T2D	T2D	M	42

Table S1 _ Elgamal et al.

Library	donor	status	RRID#	Sex	Sample Age	SampleEthnicity	DiseaseStatus	Award	TissueSource	Culture StartDate	Culture HarvestDate
HPAP-022	sample12_HPAP-022_ND	ND	RRID:SAMN19776453	F	39yo	Caucasian	No HX DIAB	UC4 DK112217	UPenn	43183	est 3/27/18
HPAP-026	sample12_HPAP-026_ND	ND	RRID:SAMN19776457	M	24yo	Caucasian	No HX DIAB	UC4 DK112217	nPod	43235	43237
HPAP-034	sample12_HPAP-034_ND	ND	RRID:SAMN19776465	M	13yo	Caucasian	No HX DIAB	UC4 DK112217	UPenn	43443	43446
HPAP-035	sample12_HPAP-035_ND	ND	RRID:SAMN19776466	M	35yo	Caucasian	No HX DIAB	UC4 DK112217	UPenn	43479	43482
HPAP-036	sample12_HPAP-036_ND	ND	RRID:SAMN19776467	F	23yo	Caucasian	No HX DIAB	UC4 DK112217	nPod	43487	43489
HPAP-037	sample12_HPAP-037_ND	ND	RRID:SAMN19776468	F	35yo	Caucasian	No HX DIAB	UC4 DK112217	UPenn	43507	43511
HPAP-039	sample12_HPAP-039_ND	ND	RRID:SAMN19776470	F	5yo	Caucasian	No HX DIAB	UC4 DK112217	nPod	43597	43601
HPAP-040	sample12_HPAP-040_ND	ND	RRID:SAMN19776471	M	35yo	Caucasian	No HX DIAB	UC4 DK112217	UPenn	43619	43623
HPAP-042	sample12_HPAP-042_ND	ND	RRID:SAMN19776473	M	13mo	Caucasian	No HX DIAB	UC4 DK112217	nPod	43630	43635
HPAP-044	sample12_HPAP-044_ND	ND	RRID:SAMN19776475	F	3yo	Caucasian	No HX DIAB	UC4 DK112217	nPod	43677	43682
HPAP-047	sample12_HPAP-047_ND	ND	RRID:SAMN19776478	M	8yo	Caucasian	No HX DIAB	UC4 DK112217	UPenn	43711	43713

HPAP-051	sample12_HPAP-051_T2D	T2D	RRID:SAMN18741940	F	43yo	African american/Black	T2DM (6 yrs duration)	U01-DK-123594	UPenn	43832	43838
HPAP-052	sample12_HPAP-052_ND	ND	RRID:SAMN19776482	M	27yo	African american/Black	No HX DIAB	U01-DK-123594	UPenn	43849	43853
HPAP-053	sample12_HPAP-053_ND	ND	RRID:SAMN19776483	F	58yo	Caucasian	No HX DIAB	U01-DK-123594	UPenn	43857	43859
HPAP-054	sample12_HPAP-054_ND	ND	RRID:SAMN19776484	F	40yo	Caucasian	No HX DIAB	U01-DK-123594	UPenn	43858	43864
HPAP-056	sample12_HPAP-056_ND	ND	RRID:SAMN19842585	M	33yo	Caucasian	No HX DIAB	U01-DK-123594	UPenn	43864	43866
HPAP-059	sample12_HPAP-059_ND	ND	RRID:SAMN19842588	M	35yo	Caucasian	No HX DIAB	U01-DK-123594	UPenn	43894	43899
HPAP-061	sample12_HPAP-061_T2D	T2D	RRID:SAMN19842590	F	59yo	African american/Black	T2DM	U01-DK-123594	UPenn	44013	44021
HPAP-063	sample12_HPAP-063_ND	ND	RRID:SAMN19842592	F	45yo	Caucasian	NO HX DIAB	U01-DK-123594	UPenn	44024	44027
HPAP-065	sample12_HPAP-065_T2D	T2D	RRID:SAMN19842594	F	54yo	Caucasian	T2DM (5 yrs)	U01-DK-123594	nPod	44032	44034
HPAP-070	sample12_HPAP-070_T2D	T2D	RRID:SAMN19842599	M	55yo	African american/Black	T2DM (15 yrs)	U01-DK-123594	nPod	44102	limited data
HPAP-074	sample12_HPAP-074_ND	ND	RRID:SAMN19842603	F	40yo	Caucasian	No Hx DIAB	U01-DK-123594	UPenn	44148	44152
HPAP-075	sample12_HPAP-075_ND	ND	RRID:SAMN19842604	M	35yo	Caucasian	No Hx DIAB	U01-DK-123594	UPenn	44158	44160

HPAP-077	sample12_HPAP-077_ND	ND	RRID:SAMN19842606	M	47yo	Caucasian	No HX DIAB	U01-DK-123594	UPenn	44168	44173
HPAP-079	sample12_HPAP-079_T2D	T2D	RRID:SAMN19842608	F	52yo	Hispanic	T2DM (6-10 yrs duration)	U01-DK-123594	nPod	44208	44209
HPAP-080	sample12_HPAP-080_ND	ND	RRID:SAMN19842609	M	22yo	Hispanic	No Hx DIAB	U01-DK-123594	nPod	44216	44218
HPAP-081	sample12_HPAP-081_T2D	T2D	RRID:SAMN19842610	F	45yo	Caucasian	T2DM (>10 yr duration)	U01-DK-123594	nPod	44223	44230
HPAP-083	sample12_HPAP-083_T2D	T2D	RRID:SAMN19842612	M	45yo	African american/Black	T2DM (2 yr duration)	U01-DK-123594	UPenn	44231	44236
HPAP-085	sample12_HPAP-085_T2D	T2D	RRID:SAMN19842614	F	48yo	Caucasian	T2DM (5-6 yr duration)	U01-DK-123594	UPenn	44243	44249
HPAP-091	sample12_HPAP-091_T2D	T2D	RRID:SAMN19842620	F	50yo	Hispanic	T2DM (<1yr)	U01-DK-123594	nPod	44300	44307
HPAP-099	sample12_HPAP-099_ND	ND	RRID:SAMN22562810	F	28yo	Hispanic	No HX DIAB	UC4-DK112217	UPenn	44371	
HPAP-100	sample12_HPAP-100_T2D	T2D	RRID:SAMN22562811	M	41yo	Caucasian	T2DM (7-8 years)	U01-DK-123594	nPod	44379	44383
HPAP-101	sample12_HPAP-101_ND	ND	RRID:SAMN22562812	F	55yo	Hispanic	No HX DIAB	U01-DK-123594	nPod	44393	44397
HPAP-103	sample12_HPAP-103_ND	ND	RRID:SAMN22562814	F	49yo	Caucasian	No HX DIAB	U01-DK-123594	UPenn	44412	44418
HPAP-104	sample12_HPAP-104_ND	ND	RRID:SAMN22562815	M	4yo	Hispanic	No HX DIAB	UC4-DK112217	UPenn	44433	44439

HPAP-105	sample12_HPAP-105_ND	ND	RRID:SAMN25600001	F	51yo	Hispanic	No HX DIAB	U01-DK-123594	nPod	44460	44462
HPAP-108	sample12_HPAP-108_T2D	T2D	RRID:SAMN25600004	M	42yo	African american/Black	T2DM	U01-DK-123594	nPod	44490	44494
HPAP-109	sample12_HPAP-109_T2D	T2D	RRID:SAMN25600005	F	59yo	Hispanic	T2DM (<5yrs)	U01-DK-123594	nPod	44512	NOT SORTED DUE TO INSUFFICIENT ISLETS

Table S1 _ Segerstolpe et al.

donor	status	Characteristics	Characteristics	Characteristics [body mass index]
sample14_H2_ND	ND	male	25	24.7
sample14_H1_ND	ND	male	43	30.8
sample14_H3_ND	ND	female	48	35
sample14_H4_ND	ND	male	22	32.9
sample14_H5_ND	ND	male	27	31.8
sample14_T2D4_T2D	T2D	female	55	29.8
sample14_T2D3_T2D	T2D	male	52	34.4

Table S1 _ Weng et al.

donor	status	HbA1C	BMI
sample17_sample17_HT1_ND	ND	5.8%	31.9
sample17_sample17_HT2_ND	ND	5.6%	24.2
sample17_sample17_HT3_ND	ND	5.4%	30.7
sample17_sample17_HT4_ND	ND	5.8%	35.1
sample17_sample17_HT5_ND	ND	5.6%	28.5
sample17_sample17_HT6_ND	ND	5.2%	23.5
sample17_sample17_HT7_ND	ND	5.4%	23.2
sample17_sample17_T2D1_T2D	T2D	6.7%	32.5
sample17_sample17_T2D2_T2D	T2D	5.9%	31.7
sample17_sample17_T2D3_T2D	T2D	7.6%	24.8
sample17_sample17_T2D4_T2D	T2D	6.7%	25.8

Table S2

Gene	Wang et al.	Camunas-Soler et al.	Xin et al. (2016)	Elgamal et al.	Segerstolpe et al.	Weng et al.	Sum	Type
DDX17	1	1	0	1	1	1	5	Up regulated
TTR	1	0	1	1	1	1	5	Down regulated
SLC30A8	1	0	0	1	1	1	4	Up regulated
TSC22D1	1	0	0	1	1	1	4	Up regulated
ALCAM	1	0	0	1	1	1	4	Up regulated
RHOBTB3	1	0	0	1	1	1	4	Up regulated
MAP2	1	1	0	1	1	0	4	Up regulated
CPE	1	0	1	0	1	1	4	Up regulated
RIN2	1	0	1	1	1	0	4	Up regulated
MEG3	1	0	0	1	1	1	4	Up regulated
NEAT1	1	0	1	1	0	1	4	Up regulated
MRAP2	1	1	0	1	1	0	4	Up regulated
KLF10	1	0	0	1	1	1	4	Up regulated
HMGB1	1	0	1	1	1	0	4	Up regulated
SSBP2	1	1	0	1	1	0	4	Up regulated
APLP2	1	0	0	1	1	1	4	Up regulated
EIF3H	1	0	0	1	1	1	4	Up regulated
PCSK1	1	0	0	1	1	1	4	Up regulated
CD47	1	0	1	1	1	0	4	Up regulated
COPA	0	1	0	1	1	1	4	Up regulated
PPP1CB	0	0	1	1	1	1	4	Up regulated
INS	1	0	0	1	1	1	4	Down regulated
MT2A	1	0	0	1	1	1	4	Down regulated
FXYD2	1	0	0	1	1	1	4	Down regulated
CCDC12	1	0	0	1	1	1	4	Down regulated
SERF2	1	0	0	1	1	1	4	Down regulated
RPL13	1	0	0	1	1	1	4	Down regulated
RPS9	1	0	0	1	1	1	4	Down regulated
RPS7	1	0	0	1	1	1	4	Down regulated
MYL6	1	0	1	1	0	1	4	Down regulated
FBXO17	1	0	0	1	1	1	4	Down regulated

Table S3 | Gene-editing of mutant cell lines

Knock-in

Cell line	Gene	No.	Mutation
Mel1 (*INS*$^{w/GFP}$)	*ZNT8*	-	ZnT8::mCherry
		-	ZnT8::RFP

Knock-out

Cell line	Gene	No.	Mutation
Mel1 (*INS*$^{w/GFP}$, *NKX6.1:2a:mC*)	*WFS1*$^{-/-}$	1	p. Pro7ArgfsLeu126*
		2	p. Arg24PhefsCys53*
HuES8	*WFS1*$^{-/-}$	-	p. Pro7ArgfsLeu126*
WFS1$^{-/-}$-1	*ZNT8*$^{-/-}$	-	p. Thr175delCys184Glyfs231*
Mel1 (*INS*$^{w/GFP}$, *ZNT8::mC*)	*WFS1*$^{-/-}$	-	p. Arg24PhefsCys53*

Table S4 _ Main Data

Dataset	Modality	Sample Size	Cell number	Year	PMID	Auther	Paper name	Raw count matrix
Cohort1	scRNA-seq	ND=12	ND (6297)	2018	29950394	Xin et al. (2018)	Pseudotime Ordering of Single Human β-Cells Reveals States of Insulin Production and Unfolded Protein Response	GSE114297
Cohort2	Patch-seq	ND=18, T2D=7	ND (227), T2D (89)	2020	32302527	Camunas-Soler et al.	Patch-Seq Links Single-Cell Transcriptomes to Human Islet Dysfunction in Diabetes	GSE12472 & GSE164875
Cohort3	scRNA-seq	ND=5	ND (10620)	2021	34428183	Shrestha et al.	Combinatorial transcription factor profiles predict mature and functional human islet α and β cells	GSE183568
Cohort4	scRNA-seq	ND=8	ND (288)	2017	28965763	Enge et al.	Single-Cell Analysis of Human Pancreas Reveals Transcriptional Signatures of Aging and Somatic Mutation Patterns	GSE81547
Cohort5	scRNA-seq	ND=5	ND (11247)	2022	36113773	Tritschler et al.	A transcriptional cross species map of pancreatic islet cells	GSE198623
Cohort6	scRNA-seq	ND=33, T2D=21	ND (24296), T2D (9902)	2023	36778506	Elgamal et al.	An integrated map of cell type-specific gene expression in pancreatic islets	http://www.gaultonlab.org/pages/Islet_expression_HPAP.html
Cohort7	scRNA-seq	ND=6, T2D=4	ND (150), T2D (130)	2016	27667667	Segerstolpe et al.	Single-Cell Transcriptome Profiling of Human Pancreatic Islets in Health and Type 2 Diabetes	https://www.ebi.ac.uk/arrayexpress/experiments/E-MTAB-5061/
Cohort8	scRNA-seq	ND=12, T2D=6	ND (117), T2D (278)	2016	27667665	Xin et al. (2016)	RNA Sequencing of Single Human Islet Cells Reveals Type 2 Diabetes Genes	Process fastq from PRJNA322072
Cohort9	scRNA-seq	ND=7, T2D=4	ND (6640), T2D (5827)	2023	37669939	Weng et al.	Single cell multiomic analysis reveals diabetes-associated β-cell heterogeneity driven by HNF1A	GSE195986
Cohort10	Multiome	ND=6, T2D=6	ND (11356), T2D (13795)	2023	37231096	Weng et al.	Integrating genetics with single-cell multiomic measurements across disease states identifies mechanisms of beta cell dysfunction in type 2 diabetes	Process fastq from PRJNA822928
Cohort11	scATAC-seq	ND=5, T2D=9	ND (21576), T2D (22739)	2023	37231096	Weng et al.		Process fastq from PRJNA716647

Table S4 _ Reguloma Data

Dataset	Modality	Antibody	Donor number	Cell type	PMID	Year	Source
dataset1	ChIP-seq	H3K27ac	4	islets	31676868	2019	PRJNA550050
dataset2	ChIP-seq	H3K27ac	1	islets	25842977	2015	PRJNA236597
dataset3	ChIP-seq	H3K27ac	1	islets	24127591	2013	PRJNA217397
dataset4	ChIP-seq	H3K4me1,H3K27ac	5	islets	30145115	2018	PRJNA317376
dataset5	HiC	-	-	beta cell	36070683	2022	PRJNA778248

Table S5 | Primers used for gene-editing and validation of mutant cell lines

Gene	Primers	
WFS1$^{-/-}$	SgRNA 1	ATTGAGTCGGGAACGCGCC
	SgRNA 2	GCGGAGCAGTGTTGGAGTC
	Validation-F	CTCCGCATGGCTCTGTTACA
	Validation-R	GCCTGCCACCCTAGTTGGTT
ZNT8$^{-/-}$	SgRNA 1	GACTGGCGTGCTAGTGTACC
	SgRNA 2	CGATGATCATCACAGTCGCC
	Validation-F	CCTTTTTGGGGGAAGTGGCAAAGT
	Validation-R	CTAACCCTGCCTCTAGCACACCAGA
ZNT8::mC ***ZNT8::RFP***	SgRNA	GTGACTGAGCTAGTCACAG
	Validation-F	AACGTGGCTTCCTCTGAGTG
	Validation-R	TACTTCGGCTCCACTCAGGA

Table S6 | Differentiation protocol

Day	Stage	Basal medium	Cytokines or Drugs	Final Con.
Day 0	**Stage 1** (Definitive Endoderm)	RPMI1640	Activin A	50 ng/ml
			Chir99021	3 μM
Day 1-2			Activin A	100 ng/ml
			SAA	50 μg/ml
			L-glutamine	1×
Day 3-5	**Stage 2** (Primitive Gut Tube)	SFD	FGF10	50 ng/ml
			Wnt3a	3 ng/ml
			MTG	4.5×10^{-4} M
			SAA	50 μg/ml
Day 6-7	**Stage 3** (Posterior Foregut)	DMEM	B27	1×
			SAA	50 μg/ml
			KAAD-cyclopamine	0.25 μM
			RA	2 μM
			NOGGIN	50 ng/ml
			FGF10	50 ng/ml
			Dorsomorphin (HuES8)	0.75 uM
Day 8-10	**Stage 4** (Pancreatic Progenitors)	DMEM	B27	1×
			SAA	50 μg/ml
			NOGGIN	50 ng/ml
			hEGF	50 ng/ml
			Nicotinamide	10 mM
Day 11-13	**Stage 5** (Endocrine Progenitors)	MCDB131	hEGF	50 ng/ml
			SANT1	0.25 μM
			LDN193189	100 nM
			RA	0.05 μM
			Alk5i-II	10 μM
			T3	1 μM
Day 14-19	**stage 6** (SC-β Cell)	MCDB131	hEGF	50 ng/ml
			LDN193189	100 nM
			GSI-XX	100 nM
			Alk5i-II	10 μM
			T3	1 μM
Day 20-33	**Stage 7** (SC-β Cell)	MCDB131	R428	2 μM
			N-Cys	1 mM
			Trolox	10 μM
			Alk5i-II	10 μM
			T3	1 μM

Table S7 | Primers used for q-PCR

	Gene	Primers	
qPCR	***WFS1***	F	CTCAACAGCTGCACCGCTGT
		R	CCATCGTGCTCGTTGACCTG
	GRP78	F	CACAGTGGTGCCTACCAAGA
		R	TGATTGTCTTTTGTCAGGGGT
	IRE1α	F	TGTTTGTCTCGACCCTGGATG
		R	CGTTGTTCTTGCCTCCAAGTG
	PERK	F	GTCCGGAACCAGACGATGAG
		R	GGCTGGATGACACCAAGGAA
	ATF6α	F	TTATCAGCATACAGCCTGCG
		R	CTTGGGACTTTGAGCCTCTG
	XBP1	F	CCTGGTTGCTGAAGAGGAGG
		R	CCATGGGGAGATGTTCTGGAG
	sXBP1	F	CTGAGTCCGCAGCAGGTG
		R	TGCCCAACAGGATATCAGACT
	ATF4	F	GACCGAAATGAGCTTCCTGA
		R	ACCCATGAGGTTTGAAGTGC
	ZNT8	F	TTGCACCAGAGATGCCTTG
		R	TCCAAGGGCATGCACAAA
	GAPDH	F	GGAGCCAAACGGGTCATCATCTC
		R	GAGGGGCCATCCACAGTCTTCT
ChIP-qPCR	***ZNT8***	F	TCGCTGCTACTTGCCAACCC
		R	TCTGACTCCTGCAAAAAGCA

Table S8 | Antibody list

	Antibody Target	Company	Cat.
ChIP	ATF4	Proteintech	10835-1-AP
WB	β-actin	Beyotime	AF1186
	eIF2α	Beyotime	AF6771
	P- eIF2α(Ser51)	Beyotime	AF1237
	ATF4	ABclonal	A25300
Staining	Insulin	DAKO	I2018
	XBP1-s	Biolegend	658802
	ZnT8	LsBio	LS-C296473
	G3BP1	Proteintech	13057-2-AP

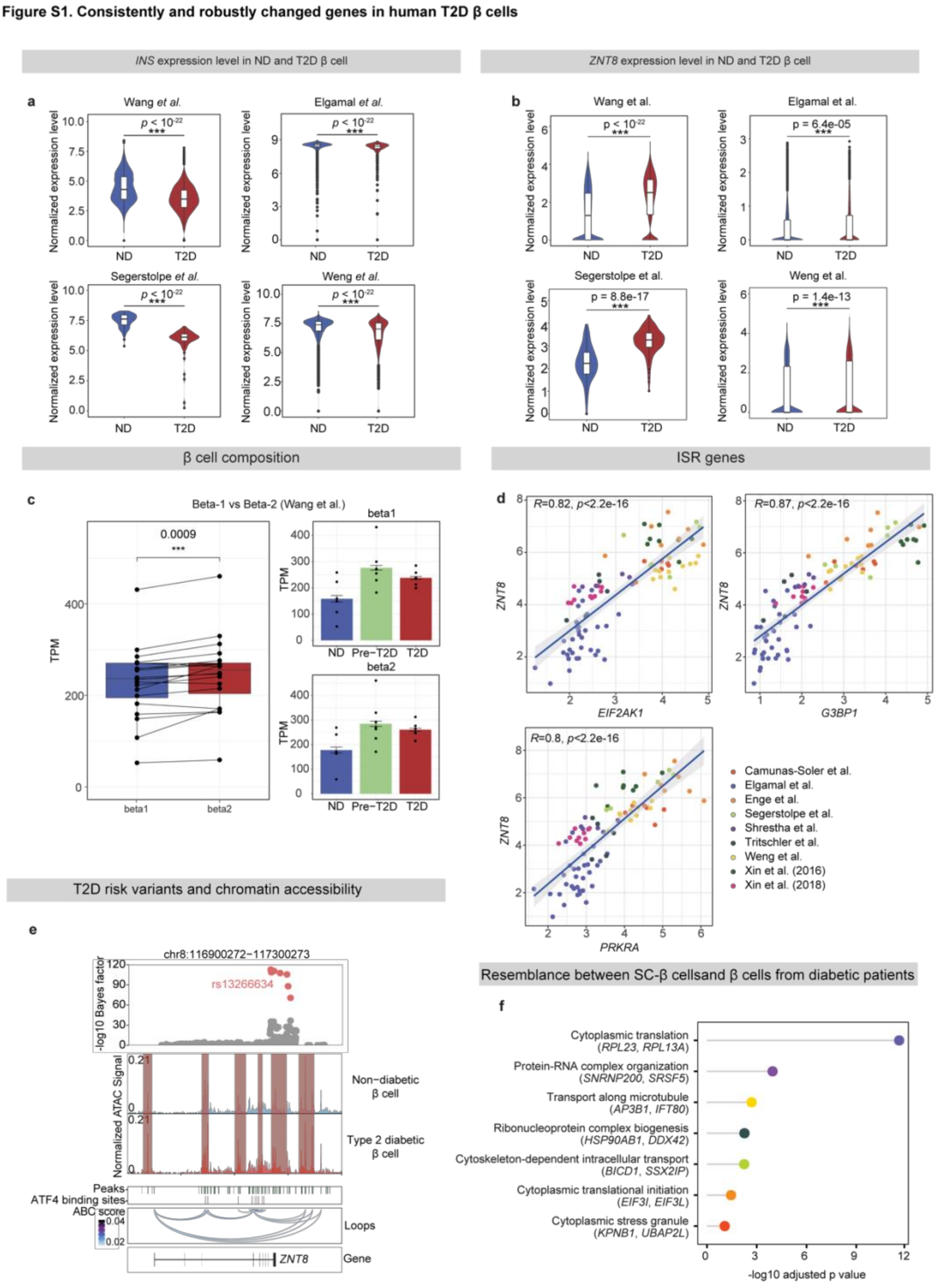

Figure S1 | Consistently and robustly changed genes in human T2D β cells.
a-b, Changes of *INS* and *ZNT8* expression level between ND and T2D β cells from independent cohorts (unpaired two-tailed *t*-tests).
c, Expression level of *ZNT8* in β cell subtypes and disease status[29] (cohort, paired two-tailed *t*-test).
d, The cis-regulatory region with differential chromatin accessibility and T2D risk variants is

highlighted in red. Chromatin accessibility and T2D GWAS data were obtained from Wang, G. et al. and the DIAMANTE (European) T2D GWAS dataset (https://kp4cd.org/node/169), respectively 1,2.

e, Pearson correlation between *ZNT8* and *EIF2AK1*, *G3BP1*, *PRKRA* expression level across donors. These donors are from distinct disease stage. Gene expression level has been transformed using ln(TPM +1).

f. GO enrichment terms and representative genes for genes consistently changed in both SC-β cells and primary human β cells.

Figure S2. *WFS1* deficiency contributes to ER stress in SC-β cells

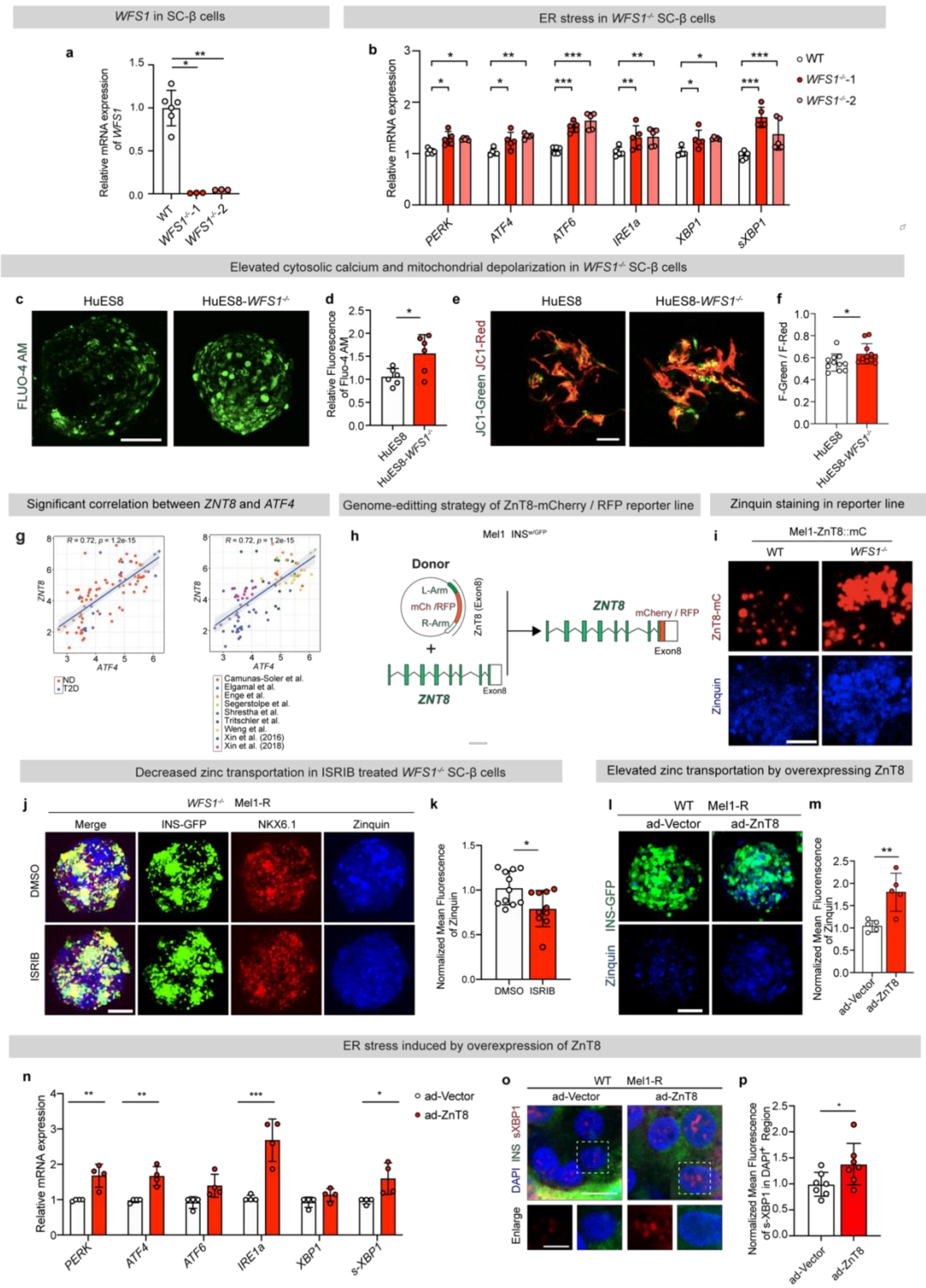

Figure S2 | *WFS1* deficiency contributes to ER stress in SC-β cells.

a, qPCR of *WFS1* in WT, *WFS1*$^{-/-}$*-1* and *WFS1*$^{-/-}$*-2*, *WFS1* expression level in knockout cell lines was normalized to WT cell lines [one-way ANOVA, n=6(WT), 3(*WFS1*$^{-/-}$*-1*), 3(*WFS1*$^{-/-}$*-2*)].

b, qPCR of ER stress related genes of Mel1-R WT, *WFS1*$^{-/-}$-1 and *WFS1*$^{-/-}$-2 SC-β cells [one-way ANOVA, n=5 of each group].

c, Representative images of fluorescence of Fluo-4 AM in HuES8-WT and HuES8-*WFS1*$^{-/-}$ SC-β

cells.

d, Quantification of mean fluorescent intensity of Fluo-4 AM in Figure S2 c (unpaired two-tailed *t*-tests, n=6 of each group) (normalized with HuES8-WT).

e, Representative images of fluorescence of JC1 in HuES8-WT and HuES8-$WFS1^{-/-}$ SC-β cells, (JC1-green, monomer; JC1-red, aggregate).

f, Quantification of the ratio of JC1-green to JC1-red in Figure S2 e (unpaired two-tailed *t*-tests, n=11 of each group) (normalized with HuES8-WT).

g, Pearson correlation between *ATF4* and *ZNT8* expression levels. Gene expression level has been transformed using ln(TPM +1).

h, Schematic diagram of constructing ZnT8::mCherry and ZnT8::RFP reporter lines in Mel1-R (INS-GFP).

i, Representative images of fluorescence with Zinquin (blue) and ZnT8::mCherry (red) in adherent single cell dissociated from WT and $WFS1^{-/-}$ ZnT8-mC SC-β cells.

j, Representative images of Zinquin fluorescence in $WFS1^{-/-}$ SC-β cells on D10 at the S7 stage treated with DMSO or 500 nM ISRIB for 24 h.

k, Quantification of mean fluorescent intensity of Zinquin in Figure S2 j [unpaired two-tailed *t*-tests, n=11(DMSO), 10 (ISRIB)] (normalized with WT group).

l, Representative images of fluorescence of Zinquin (blue) and INS-GFP (green) of WT SC-β cells transduced with ad-Vector and ad-ZnT8.

m, Quantification of mean fluorescent intensity of Zinquin in Figure S2 l (unpaired two-tailed *t*-tests, n=5 of each group) (normalized with ad-Vector transduced group).

n, qPCR of ER stress related genes of WT SC-β cells transducted with ad-Vector and ad-ZnT8 for 48 h (two-way ANOVA, n=4 of each group).

o, Representative images of immunofluorescent staining of sXBP1 (red), insulin (green) and DAPI (blue) in SC-β cells transduced with ad-Vector and ad-ZnT8.

p, Quantification of mean fluorescent intensity of sXBP1 in $DAPI^+$ region in Figure S2 o (unpaired two-tailed *t*-tests, n=7).

Scale bars, 100 μm (c, j, l), 10 μm (e, i), 5 μm (o, top row), 10μm (o, bottom row).

Data are mean ± s.d. Individual data points are shown for all bar graphs. *$P < 0.05$; **$P < 0.01$; ***$P < 0.001$; n.s., not significant.

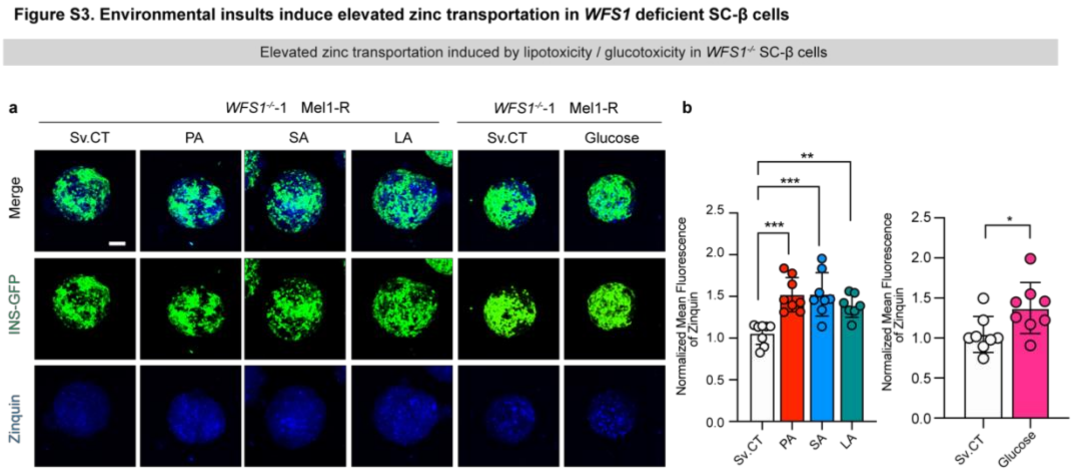

Figure S3 | Environmental insults induce elevated zinc transportation in *WFS1* deficient SC-β cells.

a, Representative images of fluorescence of Zinquin (blue) and INS-GFP (green) in Mel1-R WT SC-β cells on D10 at the S7 stage treated with vehicle or lipo- / gluco-toxicity conditions [1mM palmitic acid (PA), 0.6 mM stearic acid (SA), 0.1 mM linoleic acid (LA) and 35 mM glucose for 48 h.

b, Quantification of mean fluorescent intensity of Zinquin in Figure S3a [one-way ANOVA, n=8 (Sv.CT), 8 (PA), 8 (SA), 7 (LA), 8 (Glu)] (normalized with control group).

Scale bars, 100 μm.

Data are mean ± s.d. Individual data points are shown for all bar graphs. *P < 0.05; **P < 0.01; ***P < 0.001; n.s., not significant.

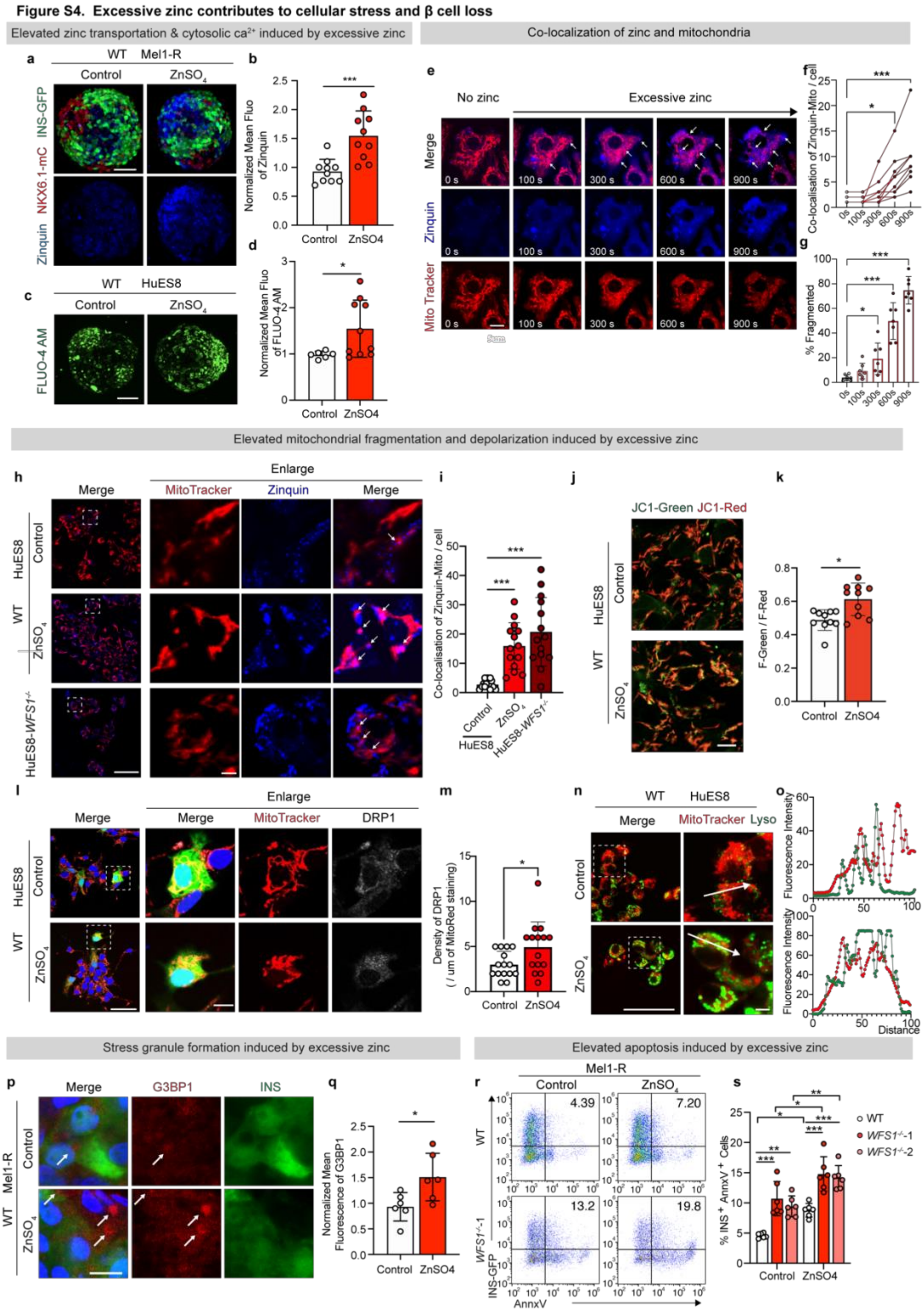

Figure S4 | Excessive zinc contributes to cellular stress and β cell loss.

a, Representative images of fluorescence of Zinquin (blue), NKX6.1-mCherry (red) and INS-GFP (green) in Mel1-R WT SC-β cells on D10 at the S7 stage treated with vehicle or 200 μM $ZnSO_4$ for 48 h.

b, Quantification of mean fluorescent intensity of Zinquin in Figure S4 a (unpaired two-tailed *t*-tests, n=10 of each group) (normalized with control group).

c, Representative images of fluorescence Fluo-4 AM in HuES8-WT SC-β cells on D10 at the S7 stage treated with vehicle and 200μM $ZnSO_4$ for 48 h.

d, Quantification of mean fluorescent intensity of Fluo-4 AM in Figure S4 c [unpaired two-tailed *t*-tests, n=6 (WT), 10 ($ZnSO_4$ treated group)] (normalized with control group).

e, Representative fluorescent images of HuES8-WT SC-β cells co-stained for Zinquin (blue) and MitoTracker Red (red). Images were taken within 900 seconds exposure to medium containing 500 μM $ZnSO_4$.

f, Co-localization of Zinquin with MitoTracker Red was calculated from data in Figure S4 e, (one-way ANOVA, n=10).

g, Percentage of cells displaying mitochondrial fragmentation following the treatments as in Figure S4 e, (one-way ANOVA, n=7).

h, Representative fluorescent images of HuES8-WT SC-β cells, wildtype SC-β cells treated with 200μM $ZnSO_4$ for 48 h and HuES8-*WFS1*$^{-/-}$ SC-β cells co-stained for Zinquin (blue) and MitoTracker Red (red).

i, Co-localization of Zinquin with MitoTracker Red was calculated from data in Figure S4 h, (one-way ANOVA, n=14).

j, Representative images of fluorescence of JC1 in HuES8-WT SC-β cells on D10 at the S7 stage treated with vehicle and 200μM $ZnSO_4$ for 48 h.

k, Quantification of the ratio of JC1-green to JC1-red in Figure S4 j (unpaired two-tailed *t*-tests, n=10 of each group) (normalized with control group).

l, Representative fluorescent images of HuES8-WT SC-β cells on D10 at the S7 stage treated with vehicle and 200μM $ZnSO_4$ for 48 h co-stained for MitoTracker Red (red), DRP1(grey) and DAPI (blue).

m, Mitochondrial Drp1 puncta density quantification (units, Drp1 puncta per μm) from data in Figure S4 l, (unpaired two-tailed *t*-tests, n=15).

n, Representative fluorescent images of HuES8-WT SC-β cells, wildtype SC-β cells treated with 200μM $ZnSO_4$ for 24 h co-stained for LysoTraker (green) and MitoTracker Red (red).

o, Percentage co-localization of LysoTraker with MitoTracker Red was calculated from data in Figure S4 n.

p, Representative images of fluorescence of G3BP1 (red), INS (green) and DAPI (blue) in adherent single cell dissociated from Mel1-R WT SC-β cell on D10 at the S7 stage treated with vehicle and 200μM $ZnSO_4$ for 48 h.

q, Quantification of mean fluorescent intensity of G3BP1 in Figure S4 p (unpaired two-tailed *t*-tests, n=6 of each group) (normalized with control group).

r, Representative flow cytometry dot plots showing the percentage of apoptotic (INS-GFP^{+}AnnxV^{+}/INS-GFP^{+}) β-cells in WT, *WFS1*$^{-/-}$-1 and *WFS1*$^{-/-}$-2 SC-β cells treated with vehicle and 50 μM $ZnSO_4$ for 48 h.

s, FACS quantification of apoptotic β-cells in WT, *WFS1*$^{-/-}$-1 and *WFS1*$^{-/-}$-2 SC-β cells treated with 50 μM $ZnSO_4$ in Figure S4 r (two-way ANOVA, n=6 of each group).

Scale bars, 100 μm (a, c), 10 μm (e, j, p); h, i, n: 50 μm (left), 5 μm (right).

Data are mean ± s.d. Individual data points are shown for all bar graphs. *P < 0.05; **P < 0.01; ***P < 0.001; n.s., not significant.

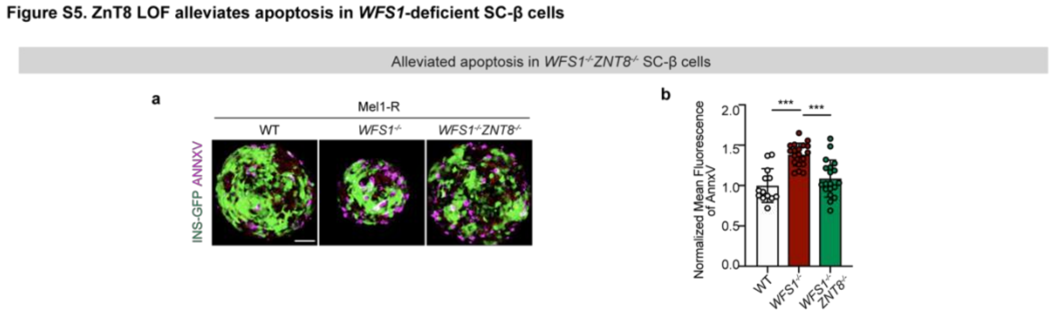

Figure S5 | ZnT8 LOF alleviates apoptosis in *WFS1*-deficient SC-β cells.

a, Representative images of fluorescence of AnnxV (magenta) and INS-GFP (green) of WT, *WFS1*$^{-/-}$ and *WFS1*$^{-/-}$*ZNT8*$^{-/-}$ SC-β cells.

b, Quantification of mean fluorescent intensity of AnnxV in Figure S5 a [one-way ANOVA, n=13(WT), 19 (*WFS1*$^{-/-}$), 19 (*WFS1*$^{-/-}$ *ZNT8*$^{-/-}$)] (normalized with WT group).

Scale bar, 100 μm (a).

Data are mean ± s.d.. Individual data points are shown for all bar graphs. *P < 0.05; **P < 0.01; ***P < 0.001; n.s., not significant.

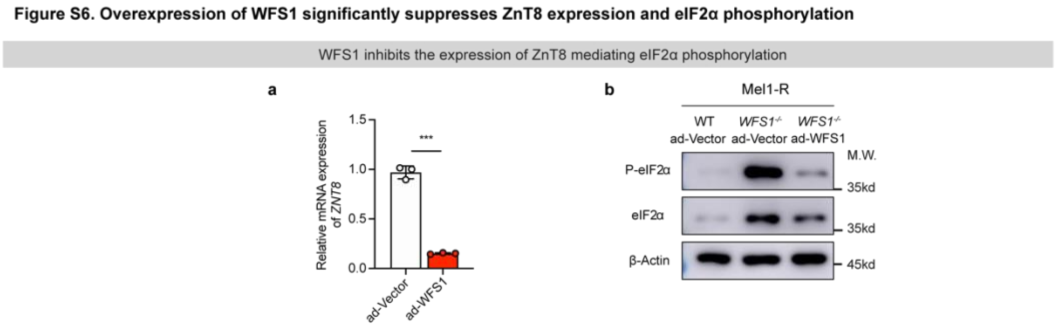

Figure S6 | Overexpression of WFS1 significantly suppresses ZnT8 expression and eIF2α phosphorylation.

a, qPCR of *ZNT8* of *WFS1*$^{-/-}$ SC-β cells transduced with ad-Vector and ad-WFS1 for 48 h (unpaired two-tailed *t*-tests, n=3 of each group).

b, Western blots showing eIF2α, P-eIF2α and β-actin of Mel-R WT and *WFS1*$^{-/-}$ SC-β cells transduced with ad-Vector and ad-WFS1 for 48 h.

Data are mean ± s.d. Individual data points are shown for all bar graphs. *P < 0.05; **P < 0.01; ***P < 0.001; n.s., not significant.

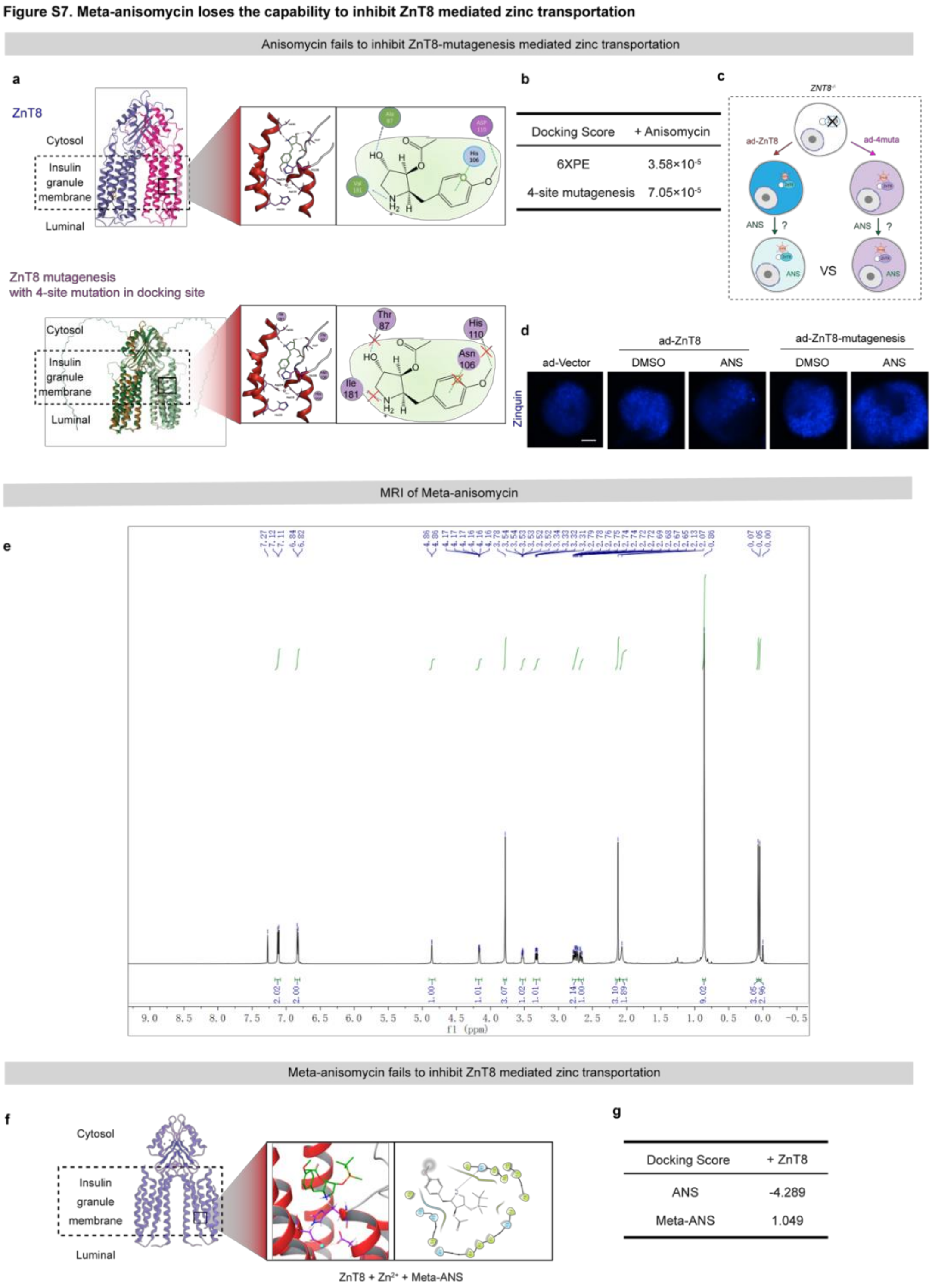

Figure S7 | Meta-anisomycin loses the capability to inhibit ZnT8 mediated zinc transportation. **a,** Interactions established after docking the Meta-ANS to ZnT8. 2D sketches show the interaction mode between ANS and residues. Molecules are green, hydrogen bonds are yellow (3D) or purple (2D). Green lines represent pipi staking and red lines represent pi-cation. The red helixes and violet sticks together form the active protein site pocket.

b, Docking scoring of ZnT8(6XPE) and ZnT8-mutagenesis with ANS.

c, Schematic diagram of overexpressing ZnT8 and ZnT8-mutagenesis in *ZNT8*$^{-/-}$ SC-β cells to confirm the inhibition of ZnT8 mediated zinc transportation by anisomycin.

d, Representative images of fluorescence of Zinquin (blue) in *ZNT8*$^{-/-}$ SC-β cells treated with 25nM DMSO or anisomycin when cultured in the presence of ad-Vector, ad-ZnT8 or ad-ZnT8-mutagenesis.

e, ^{1}H NMR spectrum of Meta-ANS [(2R,3S,4S)-4-((tert-butyldimethylsilyl)oxy)-2-(4-methoxybenzyl)pyrrolidin-3-yl acetate], ^{1}H NMR (400 MHz, CDCl3) :δ=7.11-7.12 (d, 2H), 6.82-6.84 (d, 2H), 4.86 (d, 1H), 4.17 (s,1H), 3.78 (s, 3H), 3.52-3.54 (m,1H), 3.31-3.34 (m, 1H), 2.13 (s, 3H), 2.07 (s, 2H), 0.86 (s, 9H), 0.07 (s, 3H), 0.05 (s, 3H) ppm.

f, Interactions established after docking the Meta-ANS to ZnT8. 2D sketches show the interaction mode between ANS and residues. Molecules are green, hydrogen bonds are yellow (3D) or purple (2D). Green lines represent pipi staking and red lines represent pi-cation. The red helixes and violet sticks together form the active protein site pocket.

g, Docking scoring of ANS and Meta-ANS with ZnT8(6XPE).

Scale bar, 100 μm (d).

Figure S8. Analysis of scRNA-sequencing data of SC-β cells with DMSO and anisomycin treatment

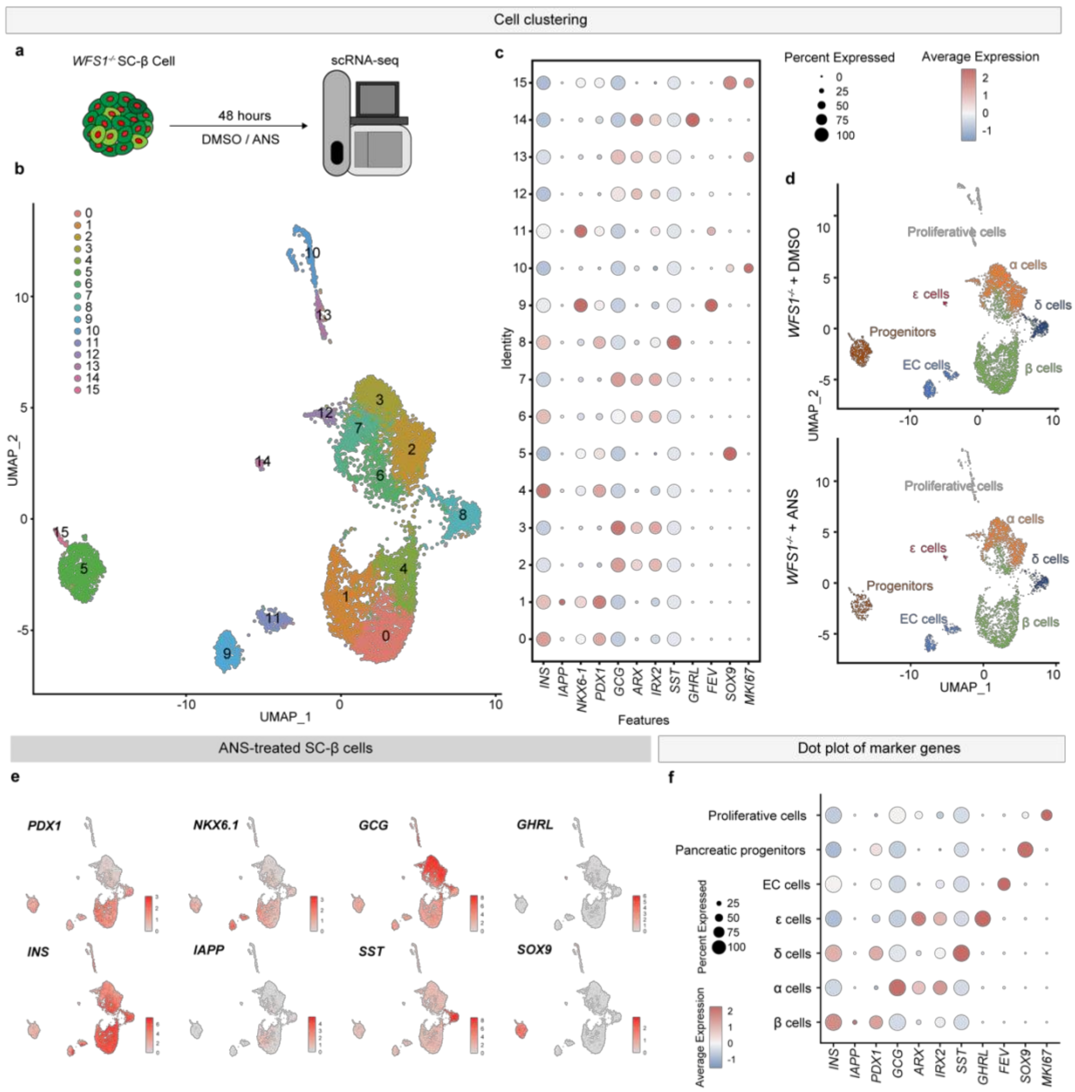

Figure S8 | Analysis of sc-RNA sequencing data of SC-β cells with DMSO and anisomycin treatment.

a, Schematic diagram of scRNA-sequencing in *WFS1*$^{-/-}$ SC-β cells treated with DMSO and anisomycin for 48 h.

b, UMAP plot of 5277 and 3329 cells from *WFS1*$^{-/-}$ SC-β cells treated with DMSO and anisomycin for 48 h, respectively. Cells are colored according to the original clusters.

c, Dot plot of gene expression levels of cell type-specific gene markers among clusters in a. Dot color shows the average expression, dot size shows the percent of cells without zero expression.

d, UMAP plot of 5277 and 3329 cells from DMSO or anisomycin treated *WFS1*$^{-/-}$ SC-β cells respectively. Cells are colored according to their assigned type.

e, UMAP plot of expression of cell type-specific gene markers.

f, Dot plot of gene expression levels of cell type-specific gene markers among cell types. Dot color shows the average expression, dot size shows the percent of cells without zero expression.

Figure S9. Low-dose anisomycin treatment suppresses ZnT8 expression

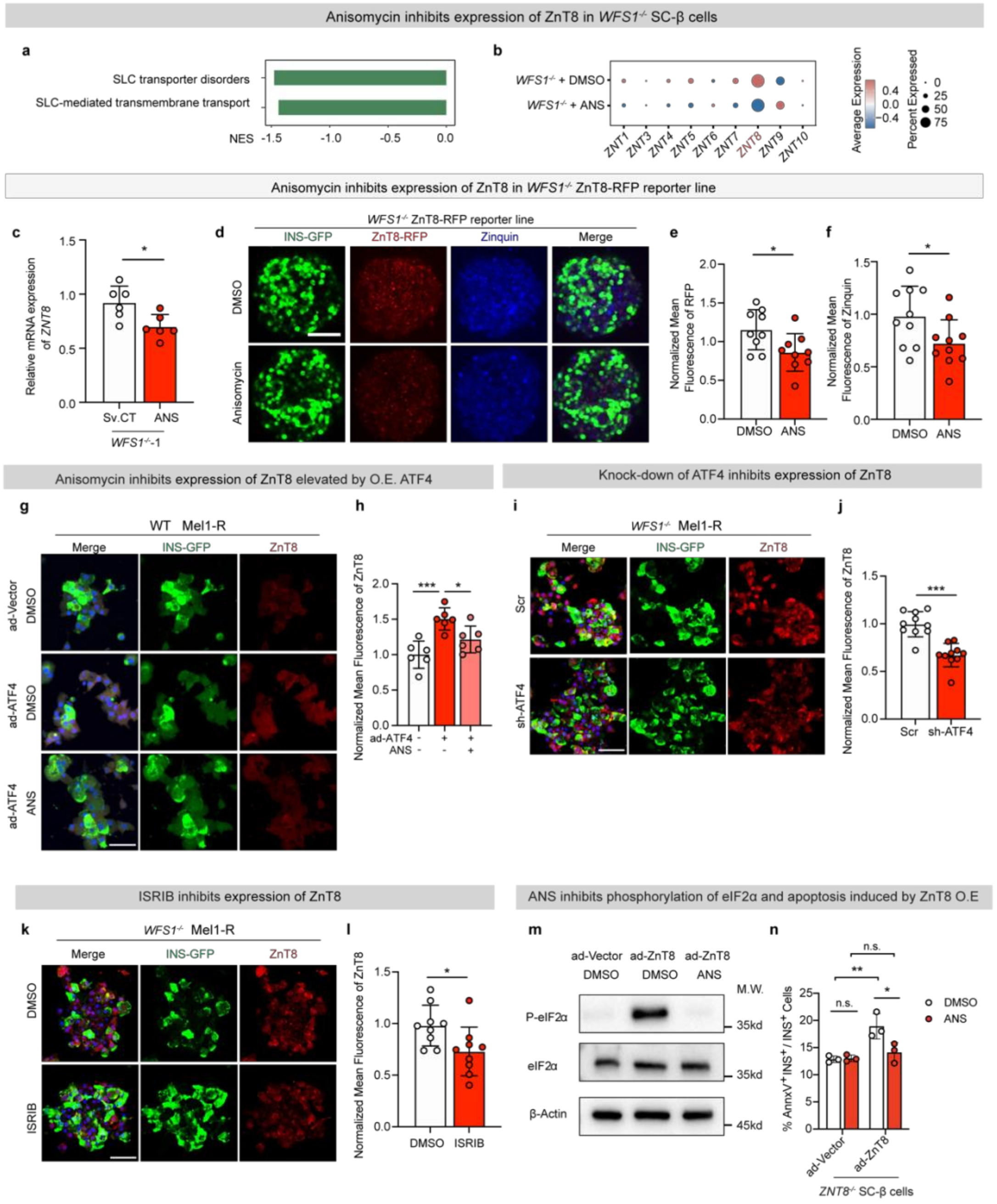

Figure S9 | Low-dose anisomycin treatment suppresses ZnT8 expression.

a, Reactome pathway enrichment analysis of differentially expressed genes between *WFS1*$^{-/-}$ SC-β cells treated with DMSO and anisomycin for 48 h using GSEA.

b, Dot plot of gene expression levels of SLC family. Dot color shows the average expression, dot size shows the percent of cells without zero expression.

c, qPCR of *ZNT8* of *WFS1*$^{-/-}$ SC-β cells treated with DMSO and anisomycin for 48 h (unpaired two-

tailed *t*-tests, n=6 of each group) (normalized with DMSO treated group).

d, Representative images of fluorescence of Zinquin (blue), ZnT8::mCherry (red) and INS-GFP (green) in *WFS1*$^{-/-}$ ZnT8-RFP SC-β cells treated with DMSO and anisomycin for 48 h.

e-f, Quantification of mean fluorescent intensity of ZnT8::RFP (e, n=9) and Zinquin (f, n=10) in FigureS9 d (normalized with DMSO treated group).

g, Representative images of fluorescence of DAPI (blue), INS-GFP (green) and ZnT8 (red) of WT SC-β cells transduced with ad-Vector and ad-ATF4 treated with Sv.CT (DMSO) and low-dose (25 nM) anisomycin for 48 h;

h, Quantification of mean fluorescent intensity of ZnT8 in Figure S9 g (one-way ANOVA, n=6) (normalized with ad-Vector transduced group).

i, Representative images of fluorescence of DAPI (blue), INS-GFP (green) and ZnT8 (red) of WFS1-/- SC-β cells transduced with Scr and sh-ATF4 treated for 72 h;

j, Quantification of mean fluorescent intensity of ZnT8 in Revision Figure S9 i (unpaired two-tailed t-tests, n=10) (normalized with ad-Vector transduced group).

k, Representative images of fluorescence of DAPI (blue), INS-GFP (green) and ZnT8 (red) of WFS1-/- SC-β cells treated with Sv.CT (DMSO) and ISRIB for 48 h;

l, Quantification of mean fluorescent intensity of ZnT8 in Revision Figure S9 k (unpaired two-tailed *t*-tests, n=9) (normalized with ad-Vector transduced group).

m, Western blots of eIF2α, P-eIF2α and β-actin of Mel-R WT SC-β cells transduced with ad-Vector and ad-ZnT8 for 48 h.

n, Flow cytometry quantification of apoptotic β-cells (two-way ANOVA, n=3 of each group).

Scale bar, 100 μm (d), 50 μm (g, i, k).

Data are mean ± s.d.. Individual data points are shown for all bar graphs. *P < 0.05; **P < 0.01; ***P < 0.001; n.s., not significant.

Figure S10. Low-dose anisomycin alleviates cellular stress in *WFS1*-deficient SC-β cells

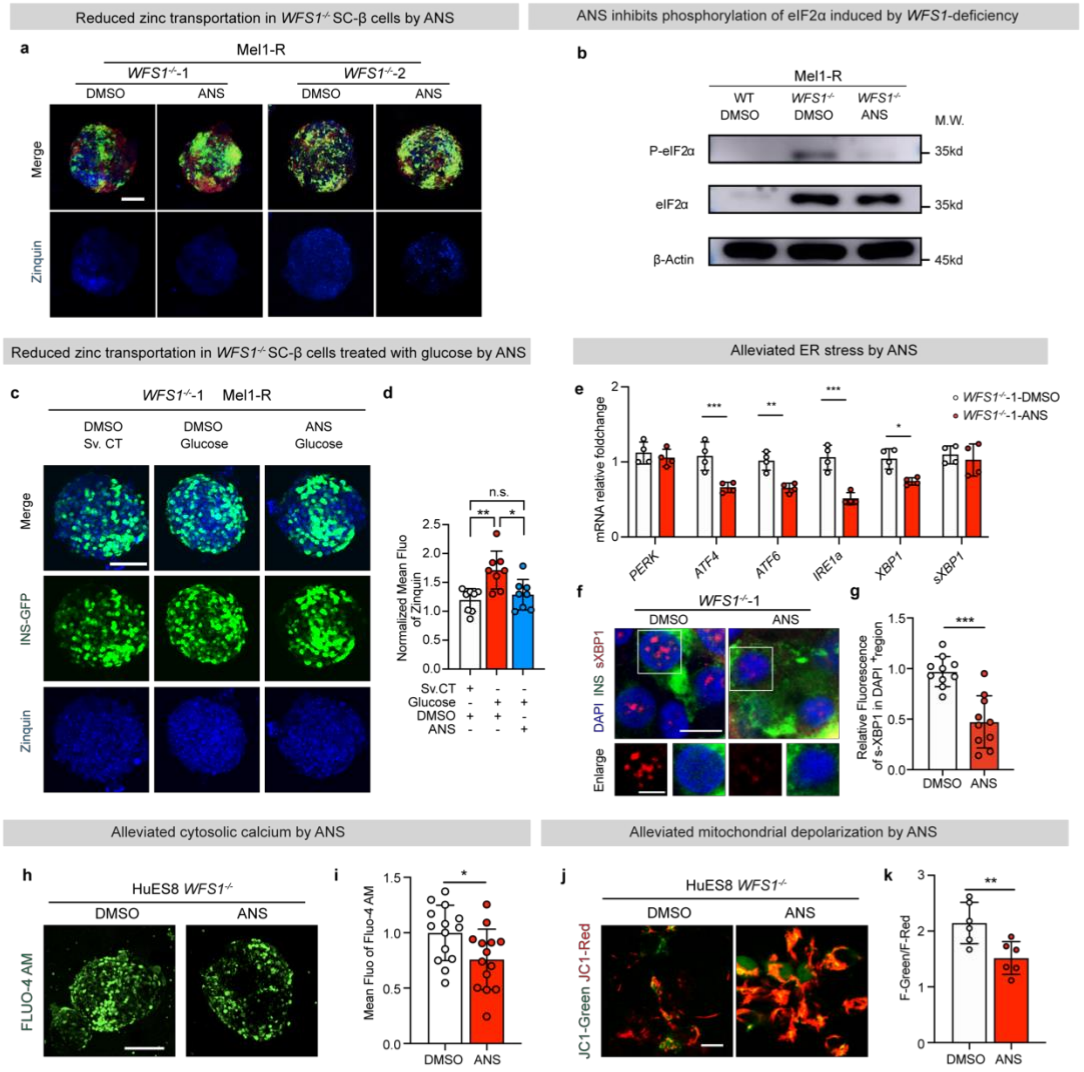

Figure S10 | Low-dose anisomycin alleviates cellular stress in *WFS1*-deficient SC-β cells.

a, Representative images of fluorescence of Zinquin (blue) in *WFS1*$^{-/-}$ SC-β cells (*WFS1*$^{-/-}$-1 and *WFS1*$^{-/-}$-2) treated with DMSO or 25 nM Anisomycin for 48 h with Zinquin (blue).

b, Western blots of eIF2α, P-eIF2α and β-actin of Mel-R WT and *WFS1*$^{-/-}$ SC-β cells treated with DMSO and 25nM anisomycin for 48 h.

c, Representative images of fluorescence of Zinquin (blue) in *WFS1*$^{-/-}$ SC-β cells treated with DMSO and 25 nM Anisomycin in solvent control or 50 mM glucose for 48 h with Zinquin (blue).

d, Quantification of mean fluorescent intensity of Zinquin in Figure S10c (One-way-ANOVA, n=8) (normalized with DMSO treated group).

e, qPCR of ER stress related genes of *WFS1*$^{-/-}$ SC-β cells treated with DMSO and 25nM anisomycin for 48 h (Two-way-ANOVA, n=4 of each group) (Normalized with DMSO treated group).

f, Representative images of immunofluorescent staining of sXBP1 (red), insulin (green) and DAPI (blue) in SC-β cells treated with DMSO and 25nM anisomycin for 48 h.

g, Quantification of mean fluorescent intensity of sXBP1 in DAPI^{+} region in Figure S10f (unpaired two-tailed *t*-tests, n=10) (normalized with DMSO treated group).

h, Representative images of fluorescence of Fluo-4 AM in HuES8-*WFS1*$^{-/-}$ SC-β cells treated with DMSO and 25nM anisomycin for 48 h.

i, Quantification of mean fluorescent intensity of Fluo-4 AM in Figure S10h (unpaired two-tailed *t*-tests, n=14 of each group) (normalized with DMSO-treated group).

j, Representative images of fluorescence of JC1 in HuES8-*WFS1*$^{-/-}$ SC-β cells treated with DMSO and 25 nM anisomycin for 48 h.

k, Quantification of the ratio of JC1-green to JC1-red in Figure S10j (unpaired two-tailed *t*-tests, n=6 of each group) (normalized with DMSO-treated group).

Scale bar=100 μm (a, c, h), 10 μm (j), 5 μm (f, bottom row), 10 μm (f, top row).

Data are mean ± s.d. Individual data points are shown for all bar graphs. *P < 0.05; **P < 0.01; ***P < 0.001; n.s., not significant.

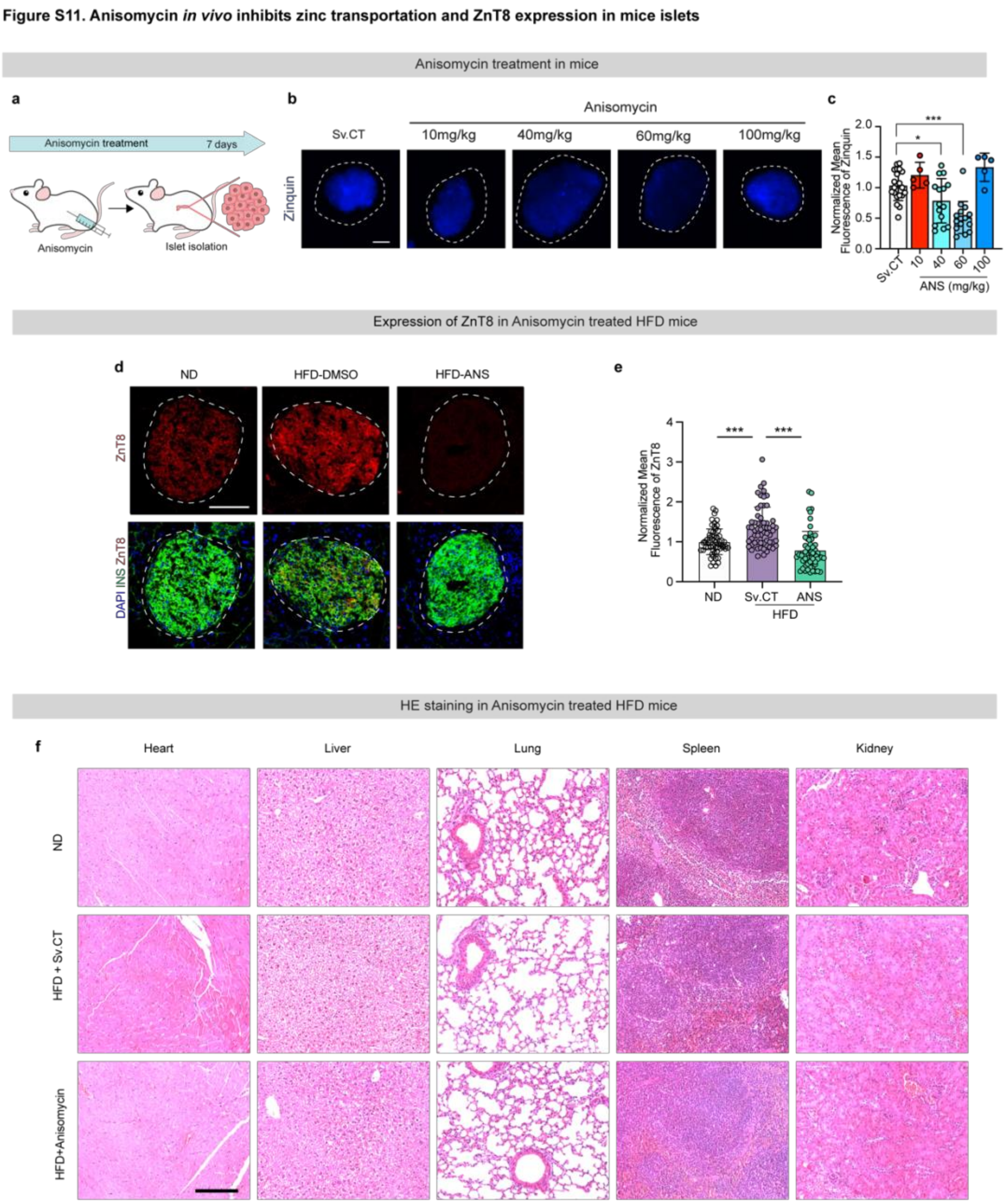

Figure S11 | Anisomycin *in vivo* inhibits zinc transportation and ZnT8 expression in mice islets.

a, Schematic diagram of therapeutic validation in anisomycin-treated C57.

b, Representative images of fluorescence of Zinquin (blue) in pancreatic islets isolated from C57 mice with Zinquin(blue), mice were treated by 10, 40, 60 and 100 mg/kg anisomycin every 48 h via i.p. injection for 7 days.

c, Quantification of mean fluorescent intensity of representative Zinquin staining images in Figure S11b [one-way-ANOVA, n= 21 (Sv.CT), 5 (10 mg/kg ANS), 15 (40 mg/kg ANS), 15 (60 mg/kg ANS), 5 (100 mg/kg ANS)] (normalized with DMSO-treated group).

d, Immunofluorescence of pancreas sections of ND mice, DMSO treated and 60 mg/kg anisomycin treated HFD-induced diabetic mice with insulin (green), ZnT8 (red) and DAPI (blue).

e, Quantification of mean fluorescent intensity of ZnT8 in Figure S11 d (one-way ANOVA, n=60) (normalized with DMSO-treated ND group). We acquired fluorescence images of representative

insulin-positive islets with a diameter greater than 50 μm, using a fixed exposure time. The expression of ZnT8 was quantified in pancreatic sections from 16-week-old male C57BL/6N mice that were fed high-fat diet and treated with Sv.CT (purple, n=5 mice) or 60 mg/kg anisomycin (green, n=5 mice) for 8 weeks, alongside age-matched C57BL/6N mice fed a normal-diet (white, n=5 mice). A total of 60 islets from each group were examined. The expression level of ZnT8 was assessed based on the mean fluorescence intensity (normalized to the area).

f, Representative H&E staining images of major organs in ND, HFD or Anisomycin treated HFD mice following i.p. GTT (n=5).

Scale bar, 100 μm (b, d), 500 μm (f)

Data are mean ± s.d. Individual data points are shown for all bar graphs. *P < 0.05; **P < 0.01; ***P < 0.001; n.s., not significant.
